# A genomic approach for accurate identification and authentication of *Alpinia galang*a (L.) Willd. using SSH and NGS technologies

**DOI:** 10.1007/s12298-026-01827-y

**Published:** 2026-10-12

**Authors:** Kannath Udayamanoharan Sanjay, Manik Vohra, Anu Radha Sharma, Chigateri M Vinay, Debyani Samantray, Vidhu Sankar Babu, Yarappa Lakshmikanth Ramachandra, Bobby Paul, Padmalatha S Rai

**Affiliations:** 1 Department of Biotechnology, Manipal School of Life Sciences, Manipal Academy of Higher Education, Manipal, India; 2 Department of Bioinformatics, Manipal School of Life Sciences, Manipal Academy of Higher Education, Manipal, India; 3 Department of Plant Sciences, Manipal School of Life Sciences, Manipal Academy of Higher Education, Manipal, India; 4 Department of Biotechnology and Bioinformatics, Kuvempu University, Jnana Sahyadri Shankaraghatta, Shivamogga, Karnataka India

**Keywords:** *Alpinia calcarata*, *Alpinia galanga*, Next generation sequencing, Species-specific DNA markers, Suppressive subtractive hybridisation, Untargeted metabolomics

## Abstract

The authenticity of herbal products is a growing global concern due to frequent mislabeling, substitution, and adulteration, especially among high-value medicinal plants. *Alpinia galanga (A.galanga)* is widely used in traditional medicine to treat various ailments. However, it is frequently substituted with *Alpinia calcarata (A.calcarata)*, which can compromise the safety and therapeutic efficacy of herbal formulations. Currently, no standardised method exists that is both rapid, sensitive, and cost-effective for distinguishing *A. galanga* from *A. calcarata.* Therefore, our study aimed to identify differential metabolites and employ suppression subtractive hybridisation (SSH) coupled with next-generation sequencing (NGS) in *A. galanga* and *A. calcarata* to develop species-specific DNA markers for the accurate authentication of *A. galanga*. The plant samples were collected and authenticated through taxonomic identification and DNA barcoding. Untargeted metabolite profiling was performed using UPLC–MS/MS. SSH, coupled with NGS, was used to develop species-specific markers, which were validated using PCR and Sanger sequencing. Taxonomic identification and DNA barcoding were successful, and the sequences were submitted to NCBI GenBank. A total of 152 metabolites were identified, and 50 significant differential metabolites were identified between *A. galanga* and *A. calcarata*, indicating chemical distinctions. The developed species-specific DNA markers demonstrated high sensitivity and specificity in distinguishing between the two species, and validation in commercial Rasna raw drug samples revealed adulteration. This study demonstrates the use of the SSH-NGS platform to develop species-specific DNA markers and provides a reliable tool for the authenticati*on of A. galanga* in both fresh plant materials and commercial products.

**Graphical Abstract:**

**Figure:**
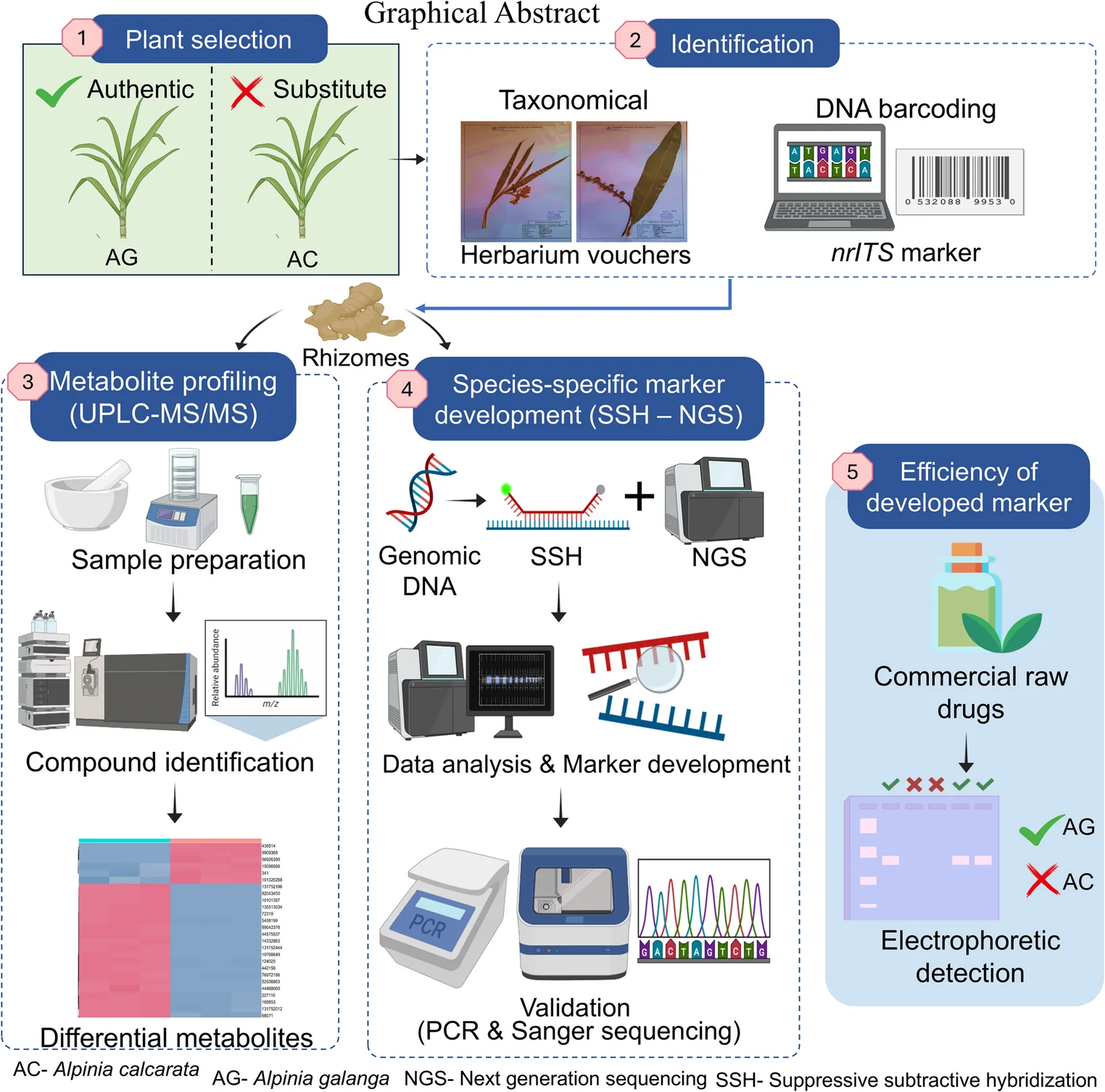

**Supplementary Information:**

The online version contains supplementary material available at https://doi.org/10.1007/s12298-026-01827-y.

## Introduction

Herbal medicines have long served as a cornerstone of healthcare, forming the basis of traditional medical systems across various cultures worldwide. In India, Ayurveda represents one of the oldest and most established traditional healing systems. Nearly 70% of the rural population depends on Ayurveda and other traditional medicine systems for primary healthcare, while the global Ayurveda market continues to expand, reflecting its growing international acceptance and use (Pandey et al. 2013; Abraham et al. 2025). This rapid commercialisation and global expansion have led to significant challenges in maintaining the authenticity and quality of medicinal plant materials. Several high-value medicinal plants are adulterated due to their morphological similarity, common vernacular name overlap, and supply-chain pressures, affecting both therapeutic efficacy and consumer safety. Studies examining the raw herbal trade have revealed adulteration rates ranging from 20% to 100%, depending on the species, geographic region, and market segment (Santhosh Kumar et al. 2018). Thus, there is a rising need for reliable, rapid, and reproducible methods to authenticate medicinal plants and ensure the integrity of herbal formulations.

*Alpinia galanga* (L.) Willd., commonly known as greater galangal or kulanjan, is a medicinal and culinary rhizome of the Zingiberaceae family that is widely used in Indian traditional medicine for treating digestive disorders, respiratory ailments, inflammatory conditions, and infectious diseases (Aziz et al. 2024). Its therapeutic properties are attributed to a complex array of bioactive compounds, including terpenoids, diarylheptanoids, flavonoids, volatile oils, and glycosides (Youn et al. 2024). It also serves as the primary botanical source for the production of Rasna, an Ayurvedic raw drug that is extensively used to treat several ailments involving inflammation, pain, and respiratory congestion (Chudiwal et al. 2010). *A. galanga* is often substituted with the rhizome of *Alpinia calcarata* (Haw.) Roscoe or *Alpinia officinarum* Hance by traders due to their shared vernacular names, similar rhizome appearance, and overlapping geographical distribution, making field identification extremely challenging for non-experts (Upadhye et al. 2018). Among these, *A. calcarata* is one of the most commonly used substitutes, presenting a significant challenge for accurate species identification. Furthermore, previous studies have reported substantial differences between *A. galanga* and *A. calcarata* in essential oil composition, phenolic content, and antioxidant potential, with *A. galanga* exhibiting higher flavonoid levels and superior bioactivity, underscoring that substituting *A. calcarata* for *A. galanga* may adversely affect therapeutic efficacy and consumer safety (Singh et al. 2020; Van et al. 2021).

Conventional identification approaches for medicinal plants are becoming increasingly inadequate for authentication, especially for closely related species such as *A. galanga* and *A. calcarata.* Morphological and microscopic examinations, although foundational to taxonomy, demand expert knowledge and are unreliable for identifying powdered or processed plant materials. Likewise, chemical fingerprinting methods, including high-performance liquid chromatography (HPLC) and mass spectrometry, can detect variations in secondary metabolite profiles but are susceptible to environmental factors, seasonal variations, cultivation practices, and post-harvest processing conditions that can significantly alter chemical signatures (Sucher and Carles 2008; Ganie et al. 2015). Although DNA barcoding offers reproducible, development-independent identification, standard universal barcodes (e.g., *rbcL*,* matK*,* ITS*) sometimes lack the resolution required to discriminate closely related congeners (Semagn et al. 2006).

Suppression Subtractive Hybridisation (SSH) is a molecular technique used to identify differences in DNA between two closely related samples by selectively enriching for sequences present in one sample but absent or less abundant in the other (Jiang 2012). When SSH is combined with next-generation sequencing (NGS), it enables efficient discovery of species-specific sequences with high discriminatory power (Choi et al. 2021). Previous studies have successfully applied this strategy for marker development in medicinal plants, including *Boerhavia diffusa* and *Tinospora cordifolia* (Sharma et al. 2023). In this study, we extend the SSH-NGS approach to develop the first species-specific DNA markers for identifying and authenticating *A. galanga.* This SSH-NGS approach exhibited high sensitivity and specificity and can be effectively extrapolated to the identification and authentication of other medicinal and economically important plant species.

## Materials and methods

### Sample collection and DNA barcoding

The medicinal plant (*A. galanga*) and its substitute (*A. calcarata*) were selected for the study from the Ayurveda Pharmacopoeia of India. Plant samples aged 10 months or older were field-collected across various seasons and locations in Karnataka, and unique accession numbers were assigned. Voucher specimens were prepared for future reference and taxonomically certified, and germplasm was preserved at the Greenhouse, Manipal School of Life Sciences (MSLS), MAHE, Manipal, India. A modified CTAB protocol was used to isolate the genomic DNA from the collected plant samples (Tiwari et al. 2012). The quality of DNA was confirmed via agarose gel electrophoresis. For DNA barcoding, PCR was performed using the universal marker *nrITS* primer (Kress et al. 2005). Following this, Sanger DNA sequencing was performed on an ABI 3500 sequencer.

### Phylogenetic and genetic distance analysis of *Alpinia spp. nrITS* sequences

Phylogenetic relationships between *Alpinia galanga*,* Alpinia calcarata*, and related congeners were evaluated using nuclear ribosomal Internal Transcribed Spacer (*nrITS*) sequences retrieved from GenBank (NCBI). The analysis included 37 *nrITS* accessions representing the target species and additional Alpinia taxa. Sequence alignment was performed with MAFFT v7.526, utilising the automatic strategy selection algorithm via the command-line interface. The resulting multiple sequence alignment was trimmed using trimAl v1.5. rev1 with the automated1 option to remove ambiguously aligned and gap-rich regions while retaining phylogenetically informative sites. The final curated alignment contained 580 nucleotide positions. Phylogenetic inference was conducted using the Maximum Likelihood (ML) method implemented in RAxML v8.2.12 (Stamatakis 2014). Tree reconstruction employed the General Time Reversible model with gamma-distributed rate heterogeneity among sites (GTRGAMMA). Nodal support was evaluated with 1,000 rapid bootstrap replicates, followed by identification of the best-scoring ML tree using the rapid bootstrap and ML search algorithm (-f a option). The resulting bootstrap-supported phylogeny was visualised and annotated using the Interactive Tree of Life (iTOL) v7 web platform. To quantify intra- vs. inter-specific divergence to support the need for species-specific markers, we constructed a pairwise per cent-identity matrix from 37 Alpinia nrITS sequences generated with Clustal Omega. Phylogenetic topology and pairwise distance analyses were jointly examined to assess species clustering patterns, identify potential anomalous accessions, and evaluate the suitability of *nrITS* as a molecular marker for distinguishing closely related species within the genus Alpinia.

### Untargeted metabolite profiling of *A. galanga* and *A. calcarata*

#### Sample preparation

Fresh field-grown rhizomes from five *A. galanga* and five *A. calcarata* plants were collected from Pilikula Nisargadhama, Mangalore, India, during July and August. The rhizomes were washed and sterilised, then dried. The dried rhizomes were powdered with liquid nitrogen. 10 mg of powder from each of the five samples was pooled to produce a composite sample for each species. 10 mg of the pooled samples were extracted with methanol (75% MeOH + 0.125% formic acid) using probe sonication for 30 min. The solution was centrifuged at 14,000 × g for 10 min at 4 °C, followed by vacuum drying the supernatant. The dried residues were reconstituted in 75% methanol and 0.125% formic acid and then filtered through a 0.22 μm syringe filter (De Vos et al. 2007). These pooled extracts were subsequently analysed in triplicate to provide technical replicates.

#### Data acquisition and data processing

The processed samples were run consecutively within a single batch under standardised instrumental conditions to reduce analytical variability. Chromatographic separation was performed using a Vanquish UHPLC system, equipped with a QExactive Orbitrap high-resolution mass spectrometer (Thermo Fisher Scientific, San Jose, CA, USA), connected to a Kinetex reverse-phase C18 column (5 μm, 250 × 4.6 mm). The flow rate was 500 µL/min, and the gradient program was set as mentioned in Vinay et al. (2025) (Vinay et al. 2025). For both MS1 and data-dependent MS/MS, the ESI ionisation source was run at 1,40,000 resolutions by switching between positive and negative ionisation modes. All metabolites within a mass range of 200–2000 m/z were obtained. Stepwise, normalised collision energies of 30, 40, and 100 units were employed for data-dependent MS/MS analysis. Thermo Xcalibur 2.0 software was used to obtain the raw mass spectrometry data (.raw files).

Following this, ProteoWizard msConvertGUI software was used to convert the raw data to .mzML format. MS-DIAL ver 4.9.2 (Tsugawa et al. 2015). was further employed to process the converted mzML centroid data in both ionisation modes via data-dependent processing. The MS-DIAL parameters were configured as follows: a minimum peak height of 30,000, a mass slice width of 0.1 Da, a sigma window value of 1.0, an MS/MS abundance cutoff of 10, a retention time tolerance of 0.05 min, and an identification score cutoff of 80% with MS1 tolerance of 0.01 and MS2 tolerance of 0.025 Da. All possible adducts for both ionisation modes were considered during peak picking and deconvolution. The final data alignment file for metabolite profiles across experimental conditions was generated using default parameters.

#### Data analysis and compound identification from MS/MS data

Multivariate analysis, including unsupervised PCA and supervised OPLS-DA, was performed between the two groups using MetaboAnalyst 6.0 (Pang et al. 2024). Cross-validation (5-fold) was also performed to assess the robustness of the PLS-DA model. Student’s t-test was performed to identify significant metabolite features. A variable importance in projection (VIP) score greater than 1.0 was used to identify metabolite abundance alterations between the groups. Metabolites exhibiting a fold change of > 1.5 or < 0.67, with a student’s *t*-test p-value and FDR-corrected p-value below 0.05, were considered significantly altered.

For metabolite annotation, precursor MS/MS ions obtained as .msp files via MS-DIAL (version 4.9.2) were exported to MS-FINDER version 3.60 (Tsugawa et al. 2015). The MS1 and MS2 tolerances were set at 0.01 and 0.05 Da, respectively. The compounds were identified using built-in databases, including HMDB, ChEBI, PlantCyc, KNApSACK, MassBank, PubChem, and NPA. Compounds identified using MS-FINDER with a confidence score greater than 5 were selected for subsequent analysis, while duplicate entries corresponding to identical compounds, retention time (RT) and m/z values were removed. Furthermore, non-phytochemical compounds were excluded by manually verifying the dataset against literature sources and online databases, retaining only phytochemicals. Finally, the selected metabolites were subjected to automated structural classification using NPClassifier (Kim et al. 2021).

### Suppressive subtractive hybridisation and next‑generation sequencing

To obtain SS markers, suppressive subtractive hybridisation coupled with NGS was performed using the PGM platform, as mentioned in Sharma et al. (2023) (Sharma et al. 2023). *A. galanga* served as the tester, and *A. calcarata* served as the driver. The enriched sequences underwent NGS library preparation using the Ion Xpress™ Plus gDNA Fragment Library Kit, following the manufacturer’s protocol with minor modifications. Tester (1 µg) and driver (2 µg) DNAs were enzymatically fragmented, with the tester DNA adapter-ligated and hybridised to driver DNA at a 1:20 ratio. The hybridised fragments were then digested using dsDNase, and the subtracted tester library was amplified with Platinum™ PCR SuperMix High Fidelity. The amplified library was enriched on the Ion OneTouch™ ES System and sequenced on the PGM platform using the Ion 316™ Chip Kit v2 BC.

#### NGS data analysis

The contigs were generated using SPAdes 3.12.0, and their quality was assessed with FastQC. The contigs with lengths > 500 bp were shortlisted, subjected to read-count analysis, and then searched for similarity using nBLAST (nucleotide Basic Local Alignment Search Tool). Contigs with mismatch percentages greater than 5 were excluded, and those with mismatch percentages of 5 or less were selected for primer designing.

### Validation of DNA markers

The primers were designed for contigs that satisfied the selection criteria after NGS data analysis. These primers were validated in 20 accessions of *A. galanga* and *A. calcarata* using PCR. Sanger sequencing was performed to confirm the specificity and accuracy of the amplified regions by using an ABI 3500 sequencer. Using NCBI BLAST, the sequence obtained from Sanger sequencing and the nodes selected for primer generation were compared for similarity. Further, the SS markers were validated in three Rasna raw drug samples collected from local vendors from different parts of India.

## Results

### Sample collection and DNA barcoding

**Fig. 1:**
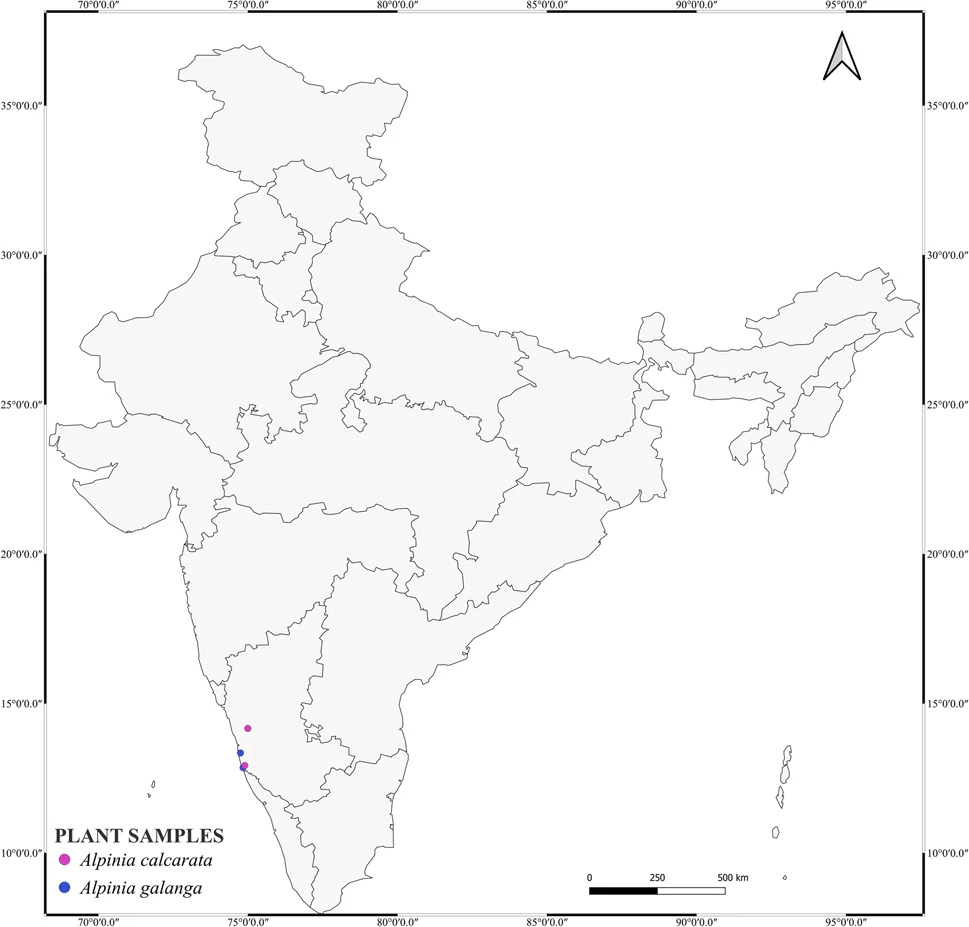
Geographical mapping of sample collection sites in southern India

The fresh plant samples *(A. galanga and A. calcarata)* used in the study were collected from various parts of Karnataka (Fig. 1) and assigned unique accession numbers (Supplementary File 1, Table S1). Herbarium voucher specimens were prepared and authenticated by a certified taxonomist (Supplementary File 1, Fig S1). The germplasm was conserved at the Greenhouse, MSLS, MAHE, Manipal (Supplementary File 1, Fig. S2). Sequences obtained after DNA barcoding with *nrITS* were submitted to GenBank NCBI (Supplementary File 1, Fig. S3), and accession IDs were obtained: ‘PP704604’ for *A.galanga* and ‘PP704605’ for *A.calcarata*.

### Phylogenetic relationships and sequence divergence among Alpinia spp

BLAST-based sequence homology of *nrITS* regions from *A. calcarata* (PP704605) used in this study share 99.43% identity with *A. galanga* (PP704604). The phylogenetic tree revealed close relationships among several Alpinia taxa, characterised by short branch lengths and limited sequence divergence (Supplementary File 1, Fig. S4). Pairwise sequence identity analysis further demonstrated high levels of sequence conservation within the genus. The mean intra-specific sequence identity was 99.48% for *A. galanga* and 99.65% for *A. calcarata*, whereas the mean inter-specific sequence identity between the two species was 99.63%. Sequence identities ranged from 99.31 to 99.66% within *A. galanga*, 99.65% within *A. calcarata*, and 99.47–99.83% between the two species (Supplementary File 2, Table S8 and S9). The substantial overlap between intra- and inter-specific sequence identity values indicates limited discriminatory resolution of the *nrITS* barcode region for unequivocal differentiation of closely related Alpinia spp.

### Untargeted metabolite profiling using UP LC-MS/MS

**Fig. 2:**
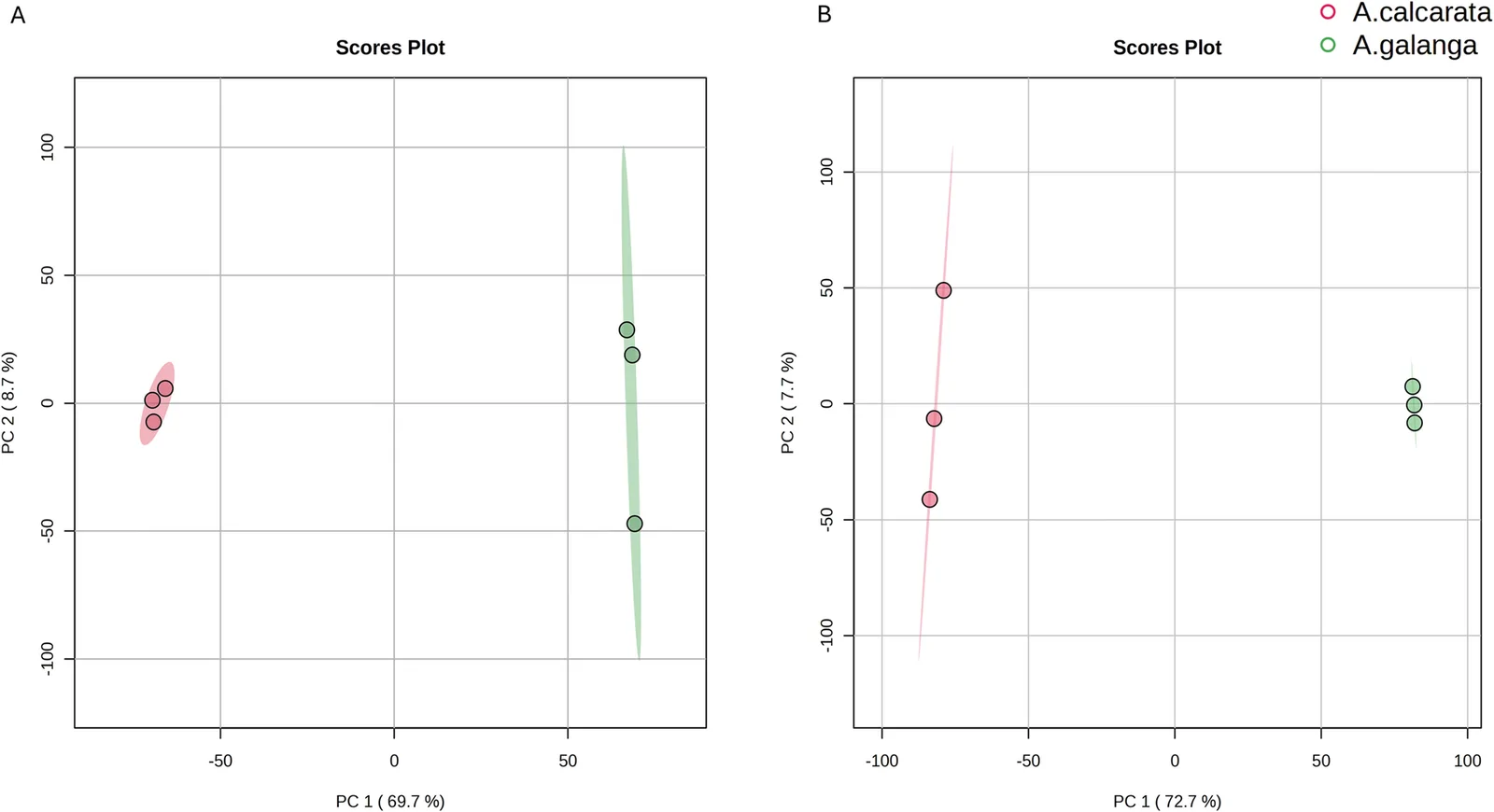
Principal component analysis (PCA) plots for LC-MS data of *A. calcarata* vs. *A. galanga*: (**A**) Positive mode (**B**) Negative mode

Metabolite profiling of methanolic rhizome extracts from *A. galanga* and *A. calcarata* was performed using UPLC–MS/MS. The total ion chromatograms are provided in Supplementary File 1, Fig. S5 and S6. Data processing and alignment in MS-DIAL (version 4.9.2) yielded 35,121 and 26,044 metabolite features in positive and negative ion modes, respectively. Unsupervised PCA analysis revealed distinct clustering of the two species across both ionisation modes (Fig. 2). OPLS-DA models were constructed with the complete normalised dataset using one predictive and one orthogonal latent variable. The supervised OPLS-DA score plot showed separation between the two samples in the positive (T score of 1: 69.6% and orthogonal T score of 7.8%) and negative (T score of 1: 72.7% and orthogonal T score of 7.7%) modes of ionisiation. R2X, R2Y, and Q2 for the fitting equations were all above 0.5, indicating that the differences among the parameters were minimal and that the equations were reliable (Supplementary File 1, Fig. S7). Student’s t-test analysis identified 4944 and 5511 significant features (*p* < 0.05) for positive and negative modes of ionisation, respectively. Following the Benjamini–Hochberg false discovery rate (FDR) correction, 4258 and 4971 remained significant (q < 0.05) in the positive and negative ionisation modes, respectively. Cross-validation analysis in MetaboAnalyst 6.0 indicated that one- and two-component models exhibited comparable predictive performance in both ionization modes. One- and two-component models showed very similar performance (1 component: Q² = 0.9739, R² = 0.9997; 2 components: Q² = 0.9741, R² = 1.0000) (Supplementary File 1, Fig. S7).

#### Screening of differential metabolites

Metabolite features showing VIP scores > 1 in both positive and negative modes were considered differentially expressed. A total of 2760 metabolite features in positive mode and 3520 in negative mode with VIP score > 1 were identified. Fold-change cutoffs of > 1.5 and < 0.67 were used to filter metabolites showing alterations between the groups. Finally, metabolite features with raw p-values and FDR-adjusted p-values below 0.05 were considered significantly differentially abundant. A total of 2079 and 2471 significantly differential metabolite features were identified in the positive and negative ionisation modes, respectively (Supplementary File 1, Table S2.

#### Metabolite annotation using UPLC‒Q-Orbitrap MS/MS data

**Fig. 3:**
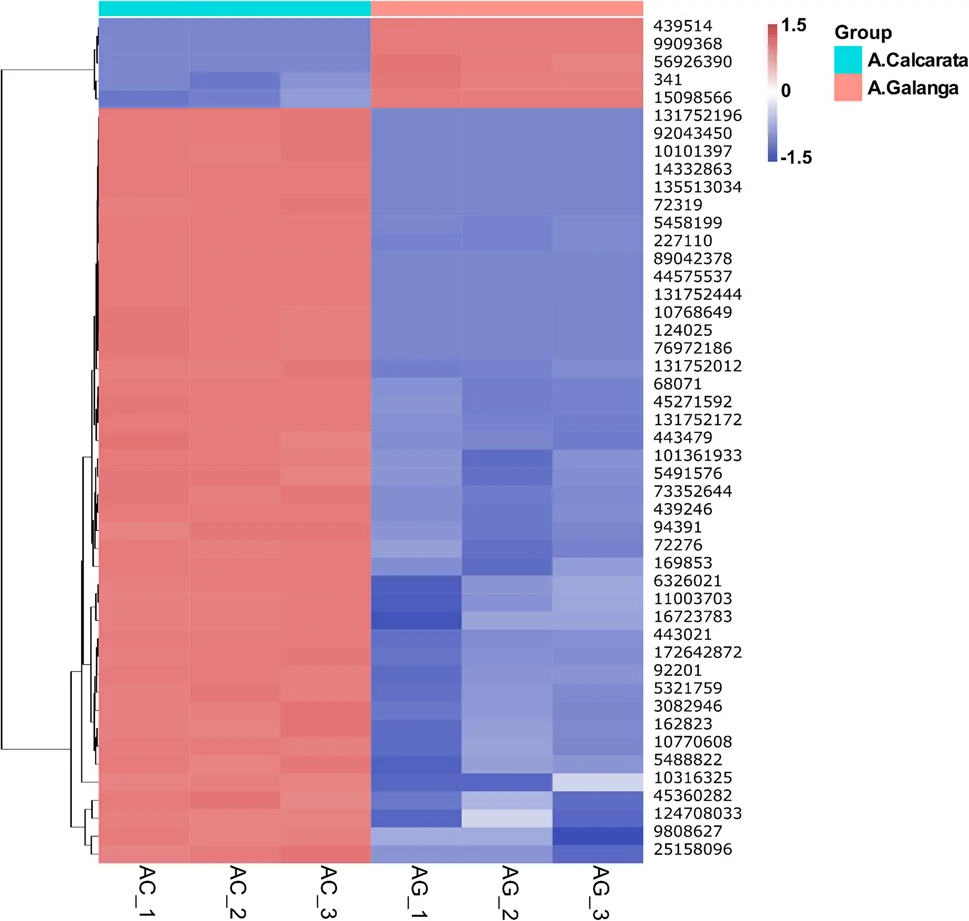
Heatmap representation of significantly differentially expressed annotated metabolites in *A. galanga* vs. *A. calcarata*

A total of 984 (positive mode) and 888 (negative mode) metabolite features with MS/MS spectrum data obtained from MS-DIAL were selected for further analysis. In silico mass spectra obtained using MS-DIAL combined with MS-FINDER software were used to match fragmentation spectra for tentative metabolite identification. 809 for positive, 619 for negative. After filtering and removing duplicate m/z and RT values, we obtained 299 for the positive ionisation mode and 348 for the negative ionisation mode. After this, the compounds were manually filtered for plant metabolites using literature searches and online databases, with a ppm error threshold of less than 10. A total of 152 compounds were structurally characterised in *A. galanga* and *A. calcarata* samples, with detailed mass spectrometry information provided in Supplementary File 1, Table S3. The major classes of annotated metabolites included flavonoids (*n* = 25), terpenoids (*n* = 33), fatty acyls (*n* = 12), and alkaloids (*n* = 12). Fifty differentially abundant metabolites were identified between the two groups by combining MS/MS data with multivariate statistical analysis in ESI positive and negative modes, as illustrated in Fig. 3 (Supplementary File 1, Table S4). The metabolite levels of scopolin increased by eighteen-fold, while ginkgolide A and glabcensin A increased by eleven-fold in *A. galanga* samples. Similarly, in *A. calcarata* the levels of clusin increased by fifteen-fold, whereas plumieride and hastatoside increased by fourteen-fold (Table 1).

**Table 1:** Details of variation in the annotated metabolite levels of *A.calcarata* vs. *A.galanga*

Metabolite Name	PubChem ID	A.calcarata	A.galanga	Fold change	Superclass	Pathway
Epicatechin	72,276	1,050,206 ± 29527.95	4090 ± 1965.1	8.0041	Flavonoids	Shikimates and Phenylpropanoids
Abietinal	443,479	15,677,711 ± 1306898.17	418,317 ± 44670.17	5.228	Diterpenoids	Terpenoids
Dehydroabietic acid	94,391	25,395,201 ± 1856300.36	748,095 ± 130053.2	5.0852	Diterpenoids	Terpenoids
Isotachioside	15,098,566	1321 ± 515.16	605,116 ± 19172.27	-8.8391	Flavonoids	Shikimates and Phenylpropanoids
3,4-dihydroxy-5-(3,4,5-trihydroxybenzoyloxy)benzoic acid	341	538,573 ± 74856.77	7,054,420 ± 284880.11	-3.7113	Aromatic polyketides	Polyketides
21-Acetyloxy-17-hydroxypregna-1,4-diene-3,20-dione	227,110	14,106 ± 1572.6	168,695 ± 12316.11	-3.58	Steroids	Terpenoids
Ginkgolide A	9,909,368	0 ± 0	177,751 ± 6676.23	-10.932	Sesquiterpenoids	Terpenoids
Myxopyroside	45,360,282	282,743 ± 24975.04	16,041 ± 6667.54	4.1396	Monoterpenoids	Terpenoids
Plumieride	72,319	2,078,015 ± 178727.34	0 ± 0	14.479	Monoterpenoids	Terpenoids
Americanin B	73,352,644	14,755,746 ± 1132196.73	83,417 ± 12145.06	7.4667	Lignans	Shikimates and Phenylpropanoids
Nomilinic acid	124,708,033	15,947,369 ± 749019.87	1262 ± 1785.21	13.557	Triterpenoids	Terpenoids
Sibiricose A6	6,326,021	973,262 ± 32953.73	11,338 ± 5381.77	6.4235	NA	Shikimates and Phenylpropanoids
Glabcensin A	10,768,649	0 ± 0	155,663 ± 67108.23	-10.74	Diterpenoids	Terpenoids
Terniflorin	131,752,172	43,319,665 ± 934705.79	42,002 ± 11546.55	10.01	Flavonoids	Shikimates and Phenylpropanoids
Ourateaproanthocyanidin A	9,808,627	393,483 ± 11385.46	16,420 ± 7672.05	4.5827	Flavonoids	Shikimates and Phenylpropanoids
thermorubin A	5,458,199	17,327,735 ± 497373.63	83,303 ± 3697.75	7.7005	Fatty acyls	Fatty acids
Pentandroside C	101,361,933	102,809,877 ± 1277489.17	9,523,115 ± 1528847.32	3.4324	Steroids	Terpenoids
Columbianetin	92,201	4,702,168 ± 414054.57	1045 ± 504.44	12.135	Coumarins	Shikimates and Phenylpropanoids
(S)-Pinocembrin	68,071	155,497,173 ± 4563198.55	29,585 ± 9498.59	12.36	Flavonoids	Shikimates and Phenylpropanoids
4-ethenylphenyl cyclopenta-1,4-diene-1-carboxylate	89,042,378	4,120,334 ± 203441.87	0 ± 0	16.515	Phenolic acids (C6-C1)	Shikimates and Phenylpropanoids
Naringenin	439,246	88,929,605 ± 3261631.5	16,813 ± 4889.16	12.369	Flavonoids	Shikimates and Phenylpropanoids
Blechnic acid	10,316,325	7,356,331 ± 505724.81	609 ± 861.73	13.492	Lignans	Shikimates and Phenylpropanoids
21-Acetyloxy-17-hydroxypregna-1,4-diene-3,20-dione	227,110	16,339,064 ± 344361.84	383,342 ± 19944.7	5.4135	Steroids	Terpenoids
evolvoid A	16,723,783	402,981 ± 17803.16	6896 ± 3287.93	5.8687	Phenylpropanoids (C6-C3)	Shikimates and Phenylpropanoids
Tortuoside	5,491,576	1,762,021 ± 148492.68	16,925 ± 4566.7	6.7019	Coumarins	Shikimates and Phenylpropanoids
Barlerin	162,823	306,880 ± 21799.32	10,152 ± 3068.33	4.9178	Monoterpenoids	Terpenoids
Clusin	44,575,537	2,064,748 ± 50627.47	0 ± 0	15.518	Lignans	Shikimates and Phenylpropanoids
Garcimangosone D	11,003,703	1,044,293 ± 49095.61	3549 ± 2019.41	8.2008	Phloroglucinols	Polyketides
Linalool 3,7-oxide beta-primeveroside	131,752,012	6,605,752 ± 550159.71	13,300 ± 2264.44	8.9562	Monoterpenoids	Terpenoids
Allanxanthone C	135,513,034	325,417 ± 14861.47	0 ± 0	12.852	Xanthones	Shikimates and Phenylpropanoids
Steganacin	443,021	1,062,116 ± 25000.45	4315 ± 1244.37	7.9434	Lignans	Shikimates and Phenylpropanoids
Icariside II	5,488,822	344,509 ± 35863.07	1220 ± 595.56	8.1407	Flavonoids	Shikimates and Phenylpropanoids
Pharboside E	45,271,592	1,923,287 ± 44541.04	26,625 ± 4797.39	6.1746	Diterpenoids	Terpenoids
Glabcensin A	10,768,649	610,870 ± 76083.85	0 ± 0	13.761	Diterpenoids	Terpenoids
Zeatin-9-glucoside-O-glucoside	172,642,872	168,007 ± 6247.83	4320 ± 788.42	5.2811	Fatty acyls	Fatty acids
Ourateaproanthocyanidin A	9,808,627	166,953 ± 10769.48	121 ± 171.12	10.117	Flavonoids	Shikimates and Phenylpropanoids
Viscumneoside VI	3,082,946	560,492 ± 61673.39	6092 ± 1515.64	6.5236	Flavonoids	Shikimates and Phenylpropanoids
Epifisetinidol-(4beta->8)-catechin	14,332,863	640,299 ± 11482.32	0 ± 0	13.829	Flavonoids	Shikimates and Phenylpropanoids
Proanthocyanidin A2	124,025	1,113,611 ± 42571.25	11,679 ± 319.87	6.5751	Flavonoids	Shikimates and Phenylpropanoids
Genistein 7-O-(2-p-coumaroylglucoside)	131,752,444	129,341,120 ± 1236310.4	335,172 ± 1043.18	8.5921	Isoflavonoids	Shikimates and Phenylpropanoids
Tinyatoxin	76,972,186	2,256,035 ± 177217.47	10,164 ± 280.61	7.7941	Diterpenoids	Terpenoids
2’’-O-p-Coumaroylvitexin	131,752,196	540,948 ± 46265.42	0 ± 0	13.586	Flavonoids	Shikimates and Phenylpropanoids
lumutinine B	56,926,390	0 ± 0	53,409 ± 11245.16	-10.245	Tryptophan alkaloids	Alkaloids
Alpindenoside A	25,158,096	338,741 ± 36405.11	4454 ± 1369.29	6.2488	Diterpenoids	Terpenoids
Taxuspine R	5,321,759	380,763 ± 24280.11	9781 ± 2345.27	5.2827	Diterpenoids	Terpenoids
Ajugaside A	10,770,608	858,928 ± 50422.12	1327 ± 761.73	9.3379	Diterpenoids	Terpenoids
Thonningianin B	10,101,397	50,043 ± 3979.29	0 ± 0	10.151	Phenolic acids (C6-C1)	Shikimates and Phenylpropanoids
Hastatoside	92,043,450	785,822 ± 70535.61	0 ± 0	14.124	Monoterpenoids	Terpenoids
Procyanidin C1	169,853	15,487,132 ± 112510.89	14,459 ± 2070.95	10.065	Flavonoids	Shikimates and Phenylpropanoids
Scopolin	439,514	0 ± 0	9,958,222 ± 565702.97	-17.788	Coumarins	Shikimates and Phenylpropanoids

### Suppressive subtractive hybridisation and next‑generation sequencing

**Fig. 4:**
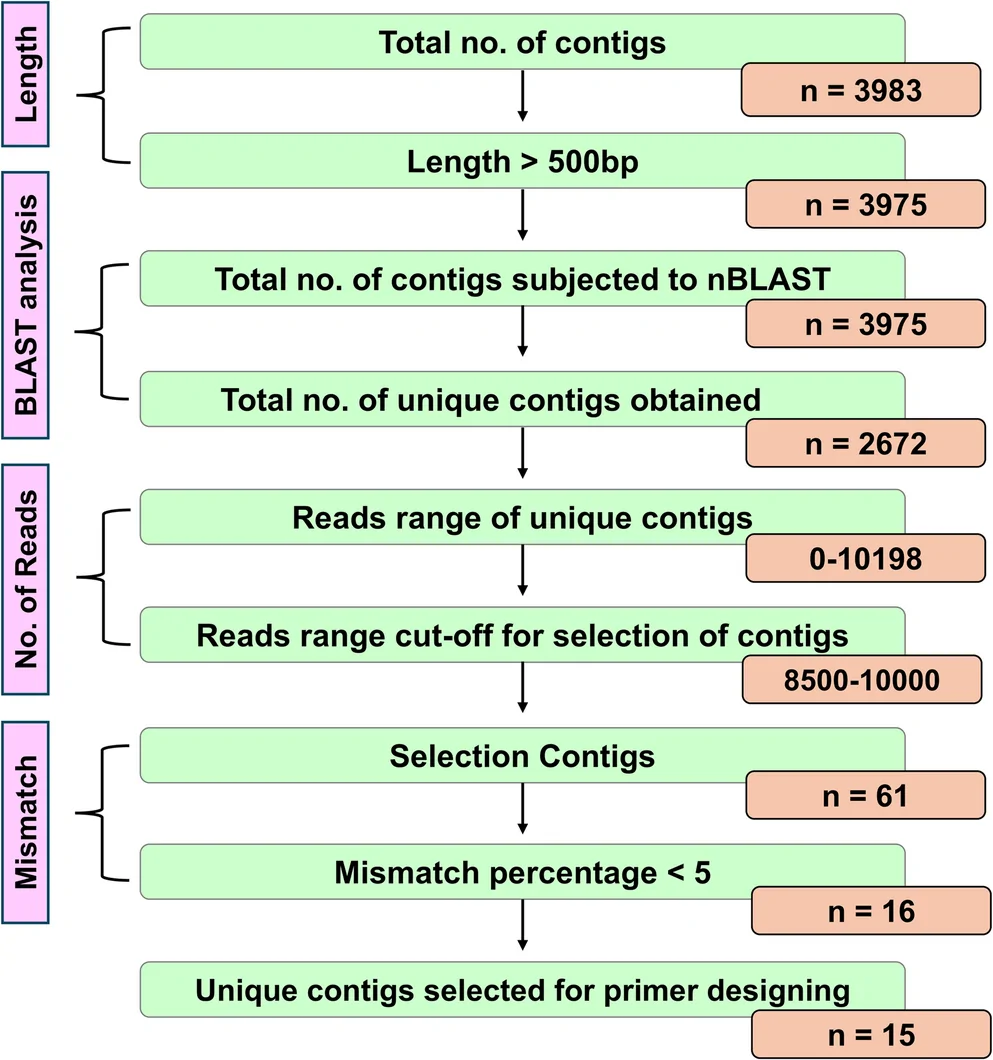
Criteria for the selection of unique contigs from the NGS data

The NGS yielded a data size of 682 MB for *A. galanga.* The details of the contigs obtained, including length, index, coverage, and GC content for the subtracted genome, are shown in Supplementary File 1, Table S5. We identified 3983 contigs after quality assessment with QUAST, and 3975 contigs with lengths > 500 bp were shortlisted and subjected to similarity search using nBLAST. In total, 2672 unique contigs with zero similarity to nucleotide sequences in the database were obtained. We obtained read counts ranging from 0 to 10,198, and contigs were filtered based on reads between 8500 and 10,000. On the basis of read counts, 61 unique contigs were selected, and the contigs with a mismatch error rate of less than five were considered for primer designing (*n* = 15). (Fig. 4).

### Validation of DNA markers

**Fig. 5:**
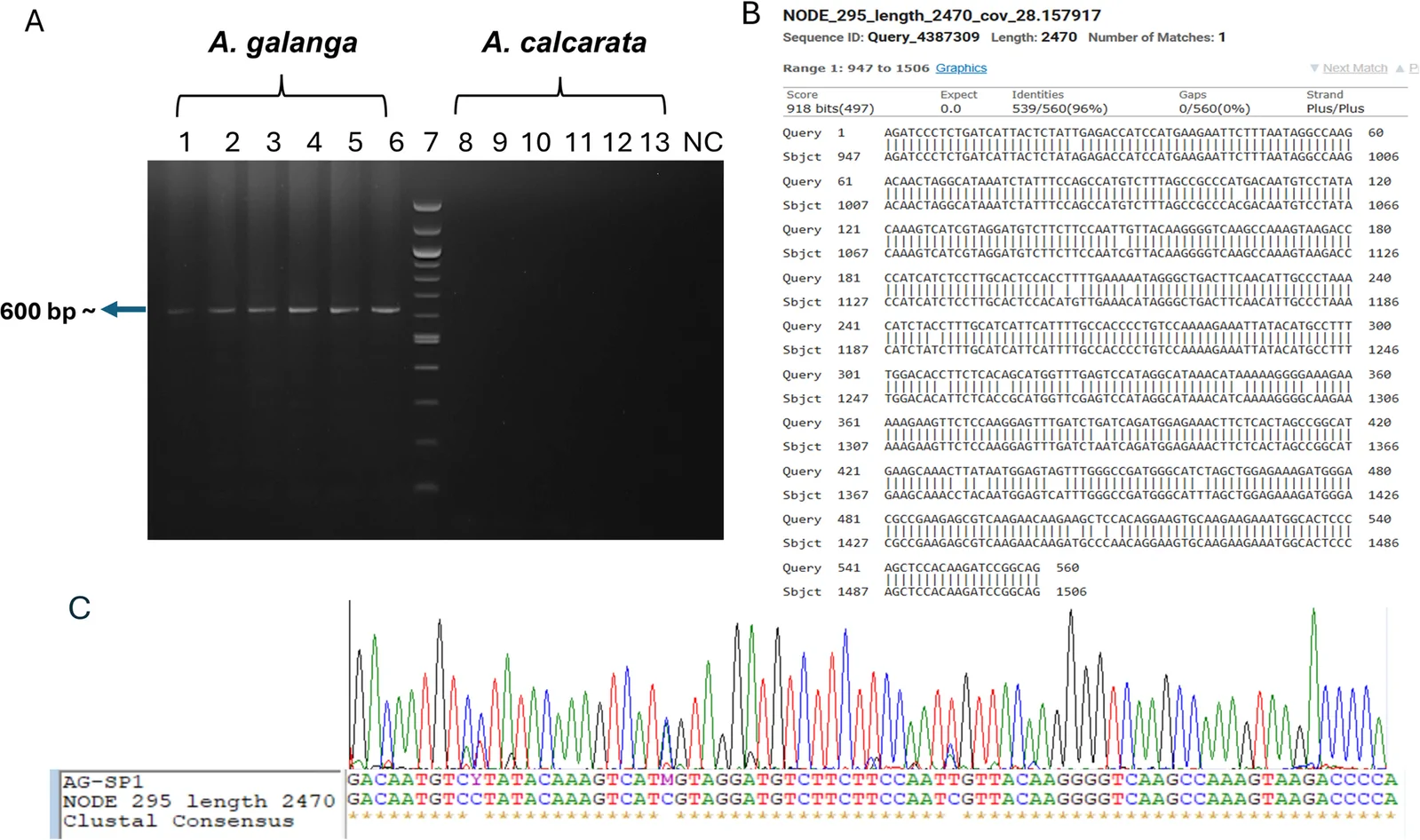
Validation of the DNA marker (AG_SP1) of *A. galanga*. (**A**) Lanes 1–6: *A. galanga* samples, 7: 100 bp DNA ladder, 8–13: *A. calcarata* samples, 14: NC (negative control), (**B**) BLAST alignment of *A. galanga* sequences matched with the original contig sequence obtained by NGS, (**C**) ClustalW alignment

A total of fifteen primer sets were designed from the shortlisted SSH-NGS contigs and screened by PCR across twenty accessions of *A. galanga* and *A. calcarata.* Among these, only one primer set showed SS amplification, while the others showed nonspecific amplification in *A. galanga* and in some *A. calcarata* samples. Only AG_SP1 could differentiate all 20 *A. galanga* accessions from 20 *A. calcarata* accessions without any false-positive or false-negative results. The observed sensitivity and specificity of the developed marker system were both 100%, with 95% confidence intervals of 83.2–100% calculated using the Clopper–Pearson exact binomial method. The electrophoretic image and Sanger sequencing results are shown in Fig. 5. The BLAST alignment of the nodes selected for designing AG_SP1 showed 98.7% identity and 100% query coverage. The SS sequences were submitted to GenBank, and the accession ID is “PP503004”(Supplementary File 1, Table S6).

**Fig. 6:**
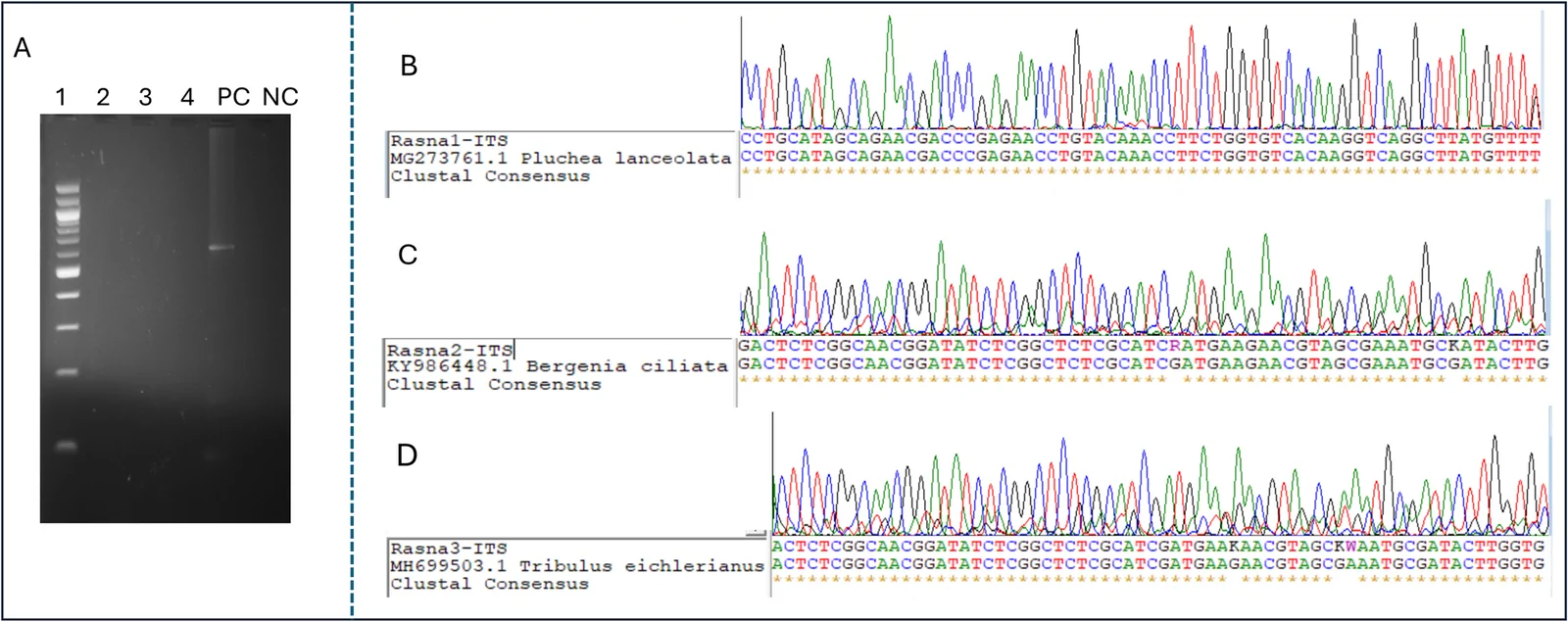
Validation of DNA marker (AG_SP1) of raw drug samples (**A**) Electrophoretic image of PCR with AG_SP1, Lane 1: Rasna 1, 2: Rasna 2, 3:Rasna 3, 4: PC (positive control), 5: (negative control). Amplification was observed in lane 4 (positive control), while no amplification was detected in lanes 1–3, indicating the absence of *A. galanga*.ClustalW alignment of *nrITS* sequences matched with NCBI GenBank of (**B**) Rasna 1, (**C**) Rasna 2, (**D**) Rasna 3

Additionally, AG_SP1 was tested on three raw Rasna drug samples (Rasna 1, Rasna 2, and Rasna 3) collected from the local vendors. No amplification was observed in any of these samples, indicating the absence of *A. galanga* in the commercially available Rasna samples (Fig. 6). To further identify the actual botanical source of Rasna samples, we performed DNA barcoding using universal marker *nrITS*, which were identified as *Pluchea Lanceolata* (Rasna 1*)*,* Bulus eichlerianus* (Rasna 2), and *Bergenia ciliataciliate* (Rasna 3), respectively. (Supplementary File 1, Table S7). These findings confirm that AG_SP1 specifically authenticates *A. galanga* and reliably excludes common substitutes or adulterants.

## Discussion

The adulteration and substitution of medicinal herbs pose significant challenges to the herbal industry, affecting therapeutic efficacy and patient safety. Although various techniques are used to prevent adulteration and authenticate medicinal plants, most lack standardisation or complete specificity. The untargeted metabolite profiling resulted in clear separation between *A. galanga* and *A. calcarata* samples in both PCA and OPLS-DA analyses, with good model performance parameters confirming that these morphologically similar species possess distinct chemical signatures. The identification of fifty significantly different metabolites provides strong biochemical evidence for species-specific biosynthetic pathways. Notably, *A. galanga* has relatively higher levels of bioactive compounds, such as scopolin and ginkgolide A, which are used for treating anti-obesity, antioxidant properties, as well as known for their anti-inflammatory properties (Geng et al. 2017; Zhang et al. 2021). In contrast, *A. calcarata* had elevated plumieride and hastatoside, which are anti-microbial, anti-diabetic and hepato-protective in nature (Du et al. 2023; El-Shiekh et al. 2024; Tomar et al. 2025).

While metabolic profiling offered insights into the distinct chemotaxonomy, chemical markers alone cannot provide reliable discrimination, as metabolite composition varies with ecological and environmental factors (Banjanac et al. 2017). In this context, the AG_SP1 marker developed in the present study effectively distinguishes *A. galanga* from closely related species such as *A. calcarata*. Validation of commercial Rasna samples confirmed the practical utility of the AG_SP1 marker. There was a complete absence of AG_SP1 amplification across all three tested market samples, and subsequent DNA barcoding identified alternative plant species, confirming genuine species substitution. These findings are consistent with prior reports of adulteration and species substitution in commercial herbal products (Santhosh Kumar et al. 2018; Ichim 2019), supporting the need for rapid and reliable authentication methods that can differentiate between closely related species.

While several DNA barcoding loci, including the internal transcribed spacer 2 (ITS2) region, have been assessed in the Zingiberaceae family, it only shows 99.5% genus-level and 73.1% species-level discrimination (Shi et al. 2011). In our results, while *nrITS* barcoding was able to resolve *A.galanga* and *A.calcarata*, the two species showed very high sequence similarity (99.43%), with substantial overlap between intra- and interspecific variation, indicating a clear absence of a barcode gap. These findings suggest that conventional barcoding regions contain only limited sequence differences between the two species, making reliable discrimination challenging. Similarly, Yang et al. (2021) identified hypervariable plastid regions that enable accurate discrimination among closely related Alpinia taxa, highlighting the limitations of conventional universal barcode loci for species-level authentication (Yang et al. 2021). The SSH-based approach used in this study acts as a genetic subtractive filter, effectively eliminating high-homology genomic backgrounds. Coupling SSH with NGS enabled targeted identification of species-specific markers without the expense of whole-genome sequencing or the low reproducibility associated with RAPD-based methods (Jiang 2012; Sahebi et al. 2015). This discovery-by-elimination process substantially reduces, though cannot entirely eliminate, the probability that the target locus is susceptible to cross-taxon amplification. Notably, only one primer set out of fifteen shortlisted genomic fragments produced discriminatory amplicons, underscoring the high sequence conservation between these sister species and the challenge of developing species-specific markers within the genus.

Overall, the developed AG_SP1 marker offers a significant practical advantage through its simplicity, speed, and cost-effectiveness, enabling authentication via a single PCR assay visualised on an agarose gel. Although the SSH-NGS pipeline used for marker discovery is relatively resource-intensive, this represents a one-time developmental step. Since the AG_SP1 established here has no higher technical demands than an endpoint PCR, it can be easily implemented in standard molecular biology laboratories without the need for sequencing or complex bioinformatics analysis. Furthermore, integrating molecular markers with metabolomic profiling can provide multilayered authentication, strengthening the traceability of plant materials. In summary, the developed AG_SP1 marker can be used not only to authenticate raw dry materials but also for identifying processed products, churnas, and polyherbal formulations where conventional methods are ineffective. Although the current assay uses a conventional thermocycler, the AG_SP1 target sequence could potentially be adapted for isothermal amplification platforms such as LAMP for portable, low-resource applications. In addition, the simple presence-or-absence readout enables straightforward interpretation and supports future development of field-deployable authentication formats.

However, despite the promising specificity of the SS markers, findings from the present study has limitations that should be considered. The analysis was performed using a limited number of samples and geographically restricted plant material, which may not fully represent the broader genetic and phytochemical diversity of Alpinia species across different regions. In addition, although AG_SP1 showed good discriminatory performance, sequence variation within the primer-binding site in genetically diverse populations cannot be ruled out and may lead to false-negative results. While the assay is technically straightforward, it was developed and validated only in a single laboratory, and external inter-laboratory validation was not performed. Furthermore, even though the marker amplified DNA from the tested commercial raw drug and churna samples, its performance in extensively processed formulations containing highly fragmented DNA or complex polyherbal matrices requires further systematic evaluation. Future studies involving larger sample sizes, broader geographic representation, multiplex marker systems, and conversion to high-throughput or field-deployable formats, such as qPCR or LAMP-based platforms, may further strengthen the translational utility of this approach for herbal authentication and quality control.

## Conclusion

The NGS-derived SS marker, AG_SP1, developed in the current study, is a cost-effective, robust, and rapid method for authenticating *A.galanga*. The marker effectively differentiated *A. galanga* from its substitute species and was validated in commercial Rasna samples, indicating its potential for routine quality control of herbal raw materials and formulations. This study presents the first report of a species-specific DNA marker for *A. galanga* validated in both authenticated plant materials and commercial products. To conclude, this strategy can be extrapolated to other medicinal plants, thereby enhancing the overall therapeutic value of herbal medicines worldwide. The development of comprehensive databases of SS sequences will aid in supporting large-scale authentication and biodiversity monitoring.

## Supplementary Information

Supplementary Material 1.

Supplementary Material 2.

Supplementary Material 3.

## Data Availability

Data will be made available on reasonable request.

## Abbreviations

SSH: Suppressive subtractive hybridisation
SS: Species-specific
NGS: Next-generation sequencing
HPLC: High-performance liquid chromatography
nrITS: Nuclear ribosomal internal transcribed spacer
LC-MS: Liquid chromatography–mass spectrometry
PCA: Principal component analysis
