# Supplementary material for: A genomic approach for accurate identification and authentication of Alpinia galanga (L.) Willd. using SSH and NGS technologies
Source: Physiol Mol Biol Plants. 2026 Oct 12;32(11):2479–91. doi: 10.1007/s12298-026-01827-y (PMC13759926; doi:10.1007/s12298-026-01827-y)
Supplement: Supplementary file 1 [file 12298_2026_1827_MOESM1_ESM.docx]

**Table S1** Details of *Alpinia galanga* and *Alpinia calcarata* plant collection

| **Sl. No.** | **Accession number** | **Plant Name** | **Collection material** | **Stored/Grown** | **Herbarium voucher numbers** |
| --- | --- | --- | --- | --- | --- |
| 1 | 20200027A | *A. galanga* | Rhizhome | Grown | MSLS-AG-01 |
| 2 | 20200027B | *A. galanga* | Rhizhome | Grown | MSLS-AG-02 |
| 3 | 20200027C | *A. galanga* | Rhizhome | Grown | MSLS-AG-03 |
| 4 | 20200027D | *A. galanga* | Rhizhome | Grown | MSLS-AG-04 |
| 5 | 20200027E | *A. galanga* | Rhizhome | Grown | MSLS-AG-05 |
| 6 | 20200027F | *A. galanga* | Rhizhome | Grown | MSLS-AG-06 |
| 7 | 20200027G | *A. galanga* | Rhizhome | Grown | MSLS-AG-07 |
| 8 | 20200027H | *A. galanga* | Rhizhome | Grown | MSLS-AG-08 |
| 9 | 20200027I | *A. galanga* | Rhizhome | Grown | MSLS-AG-09 |
| 9 | 20200027J | *A. galanga* | Rhizhome | Grown | MSLS-AG-10 |
| 10 | 20200027K | *A. galanga* | Rhizhome | Grown | MSLS-AG-11 |
| 11 | 20210068A | *A. galanga* | Rhizhome | Grown | MSLS-AG-12 |
| 12 | 20210068B | *A. galanga* | Rhizhome | Grown | MSLS-AG-13 |
| 13 | 20220069A | *A. galanga* | Rhizhome | Grown | MSLS-AG-14 |
| 14 | 20220069B | *A. galanga* | Rhizhome | Grown | MSLS-AG-15 |
| 15 | 20220074A | *A. galanga* | Rhizhome | Grown | MSLS-AG-16 |
| 16 | 20220075A | *A. galanga* | Rhizhome | Grown | MSLS-AG-17 |
| 18 | 20220075B | *A. galanga* | Rhizhome | Grown | MSLS-AG-18 |
| 19 | 202200876A | *A. galanga* | Rhizhome | Grown | MSLS-AG-19 |
| 20 | 20220076B | *A. galanga* | Rhizhome | Grown | MSLS-AG-20 |
| 21 | 20210028A | *A. calcarata* | Rhizhome | Grown | MSLS-AC-01 |
| 22 | 20210028B | *A. calcarata* | Rhizhome | Grown | MSLS-AC-02 |
| 23 | 20210028C | *A. calcarata* | Rhizhome | Grown | MSLS-AC-03 |
| 24 | 20210028D | *A. calcarata* | Rhizhome | Grown | MSLS-AC-04 |
| 25 | 20210028E | *A. calcarata* | Rhizhome | Grown | MSLS-AC-05 |
| 26 | 20210028F | *A. calcarata* | Rhizhome | Grown | MSLS-AC-06 |
| 27 | 20210028G | *A. calcarata* | Rhizhome | Grown | MSLS-AC-07 |
| 28 | 20210028H | *A. calcarata* | Rhizhome | Grown | MSLS-AC-08 |
| 29 | 20210028I | *A. calcarata* | Rhizhome | Grown | MSLS-AC-09 |
| 30 | 20210028J | *A. calcarata* | Rhizhome | Grown | MSLS-AC-10 |
| 31 | 20210028K | *A. calcarata* | Rhizhome | Grown | MSLS-AC-11 |
| 32 | 20210029A | *A. calcarata* | Rhizhome | Grown | MSLS-AC-12 |
| 33 | 20210029B | *A. calcarata* | Rhizhome | Grown | MSLS-AC-13 |
| 34 | 20220090A | *A. calcarata* | Rhizhome | Grown | MSLS-AC-14 |
| 35 | 20220090B | *A. calcarata* | Rhizhome | Grown | MSLS-AC-15 |
| 36 | 20220091A | *A. calcarata* | Rhizhome | Grown | MSLS-AC-16 |
| 37 | 20220091B | *A. calcarata* | Rhizhome | Grown | MSLS-AC-17 |
| 38 | 20220091C | *A. calcarata* | Rhizhome | Grown | MSLS-AC-18 |
| 39 | 20220092A | *A. calcarata* | Rhizhome | Grown | MSLS-AC-19 |
| 40 | 20220092A | *A. calcarata* | Rhizhome | Grown | MSLS-AC-20 |

**Table S2** Summary of the metabolite filtering workflow, culminating in the identification of key differential metabolites

|  | **No of features (Positive mode)** | **No of features (Negative mode)** |
| --- | --- | --- |
| **VIP>1** | 2760 | 3520 |
| **FC (>1.5 or <0.67)** | 2215 | 2471 |
| **P value < 0.05** | 2079 | 2471 |
| **FDR corrected (q<0.05)** | 2079 | 2471 |
| **Annotated metabolites (MS/MS)** | 60 | 92 |
| **Differential metabolites** | 17 | 33 |
| **Total differential metabolites** | 50 | |

**Table S3** Tentaive compound identification of *A.calcarata* and *A.galanga* by UPLC‒Q-Orbitrap MS/MS in positive and negative modes of ionisation

|  | **Putative compound name** | **Retention time** | **Observed m/z** | **Precursor m/z** | **PPM error** | **Adduct** | **MS/MS fragments** | **PubChem ID** | **Class** | **Pathway** |
| --- | --- | --- | --- | --- | --- | --- | --- | --- | --- | --- |
| 1. | Sebacic acid | 25.13 | 201.1132 | 201.1119 | -6.62836 | [M-H]- | 57.033, 139.1113, 201.112 | 5192 | Dicarboxylic acids | Fatty acids |
| 2. | L-Galacto-2-heptulose | 5.22 | 209.0667 | 209.0653 | -6.58536 | [M-H]- | 85.0278, 209.0655 | 548228 | Monosaccharides | Carbohydrates |
| 3. | Ichthyotherol | 24.69 | 213.0921 | 213.0909 | -5.64866 | [M-H]- | 122.0354, 213.0904 | 6451387 | NA | NA |
| 4. | Isocitric acid | 10.3 | 215.0162 | 215.0156 | -2.88625 | [M+Na]+ | 95.0858, 169.0106, 215.0158 | 1198 | Dicarboxylic acids | Fatty acids |
| 5. | Solavetivone | 25 | 219.1743 | 219.1737 | -2.92608 | [M+H]+ | 53.0392, 95.0494, 219.1737 | 442399 | Spirovetivane sesquiterpenoids | Terpenoids |
| 6. | Cis-zeatin | 18.24 | 220.1193 | 220.1175 | -8.11406 | [M+H]+ | 56.9654, 90.0552, 116.0342, 151.0275, 202.1069 | 688597 | Purine alkaloids | Alkaloids |
| 7. | Alchorneine | 30.05 | 220.1455 | 220.1456 | 0.289636 | [M-H]- | 93.0329, 148.0515, 220.1459 | 217611 | Imidazole alkaloids | Alkaloids |
| 8. | Carbofuran | 5.26 | 222.1125 | 222.1118 | -3.01352 | [M+H]+ | 58.0658, 91.0546, 119.0491, 178.1223, 222.112 | 2566 | NA | NA |
| 9. | Anaferine | 27.19 | 225.1961 | 225.1956 | -2.39521 | [M+H]+ | 55.0549, 100.1123, 143.1177, 225.1956 | 443143 | Piperidine alkaloids | Alkaloids |
| 10. | (1R,4S)-1-Hydroperoxy-p-menth-2-en-8-ol acetate | 26.45 | 227.1289 | 227.1277 | -5.20898 | [M-H]- | 107.0485, 227.1067 | 10889657 | Menthane monoterpenoids | Terpenoids |
| 11. | Sepiapterin | 5.13 | 236.0789 | 236.0766 | -9.79855 | [M-H]- | 74.023,116.0333 | 1.35E+08 | pteridine alkaloids | Alkaloids |
| 12. | Kigelinone | 27.92 | 239.0344 | 239.0339 | -2.23156 | [M-H2O-H]- | 102.9551, 211.039, 239.0342 | 442752 | Naphthoquinones | Polyketides |
| 13. | 1,11-Undecanedicarboxylic acid | 27.49 | 243.1602 | 243.1591 | -4.45484 | [M-H]- | 243.1592 | 10458 | Dicarboxylic acids | Fatty acids |
| 14. | Columbianetin | 20.43 | 245.0819 | 245.0809 | -4.21459 | [M-H]- | 109.0277, 203.0703, 245.0812 | 92201 | Furocoumarins | Shikimates and Phenylpropanoids |
| 15. | Ruspolinone | 25.83 | 248.1292 | 248.1291 | -0.47342 | [M-H]- | 70.0282, 248.129 | 442655 | Piperidine alkaloids | Alkaloids |
| 16. | Tuberostemospironine | 16.81 | 254.1387 | 276.1201 | -1.90483 | [M+Na]+ | 91.0546, 217.0467, 276.12 | 1.01E+08 | Stemona alkaloids | Alkaloids |
| 17. | (S)-Pinocembrin | 26.97 | 255.0663 | 255.0654 | -3.4613 | [M-H]- | 151.0022, 213.0548, 255.0657 | 68071 | Flavanones | Shikimates and Phenylpropanoids |
| 18. | 4-ethenylphenyl cyclopenta-1,4-diene-1-carboxylate | 24.69 | 257.0819 | 257.0812 | -2.84198 | [M+FA-H]- | 122.0354 | 89042378 | Bagremycins | Shikimates and Phenylpropanoids |
| 19. | Naringenin | 25.22 | 271.0612 | 271.0604 | -2.94207 | [M-H]- | 125.0227, 271.0608 | 439246 | Flavanones | Shikimates and Phenylpropanoids |
| 20. | Phenylglucuronide | 4.73 | 271.0812 | 271.0804 | -3.05731 | [M+H]+ | 97.0286, 193.0493, 235.0595, 271.0805 | 119239 | Simple phenolic acids | Shikimates and Phenylpropanoids |
| 21. | nonadecan-2-amine | 27.36 | 284.3312 | 284.3304 | -2.73031 | [M+H]+ |  | 141647 | Hydrocarbons | Fatty acids |
| 22. | salicyl-6-hydroxy-2-cyclohexene-on-oyl | 26.14 | 285.0733 | 245.0779 | 5.817661 | [M+H-H2O]+ | 90.0553, 171.0049, 245.078 | 14731723 | Simple phenolic acids | Shikimates and Phenylpropanoids |
| 23. | Homoarbutin | 16.22 | 285.098 | 285.0978 | -0.62052 | [M-H]- | 109.0305, 122.0356 | 324796 | NA | NA |
| 24. | Abietinal | 30.61 | 287.2369 | 287.2361 | -2.9299 | [M+H]+ | 149.1321, 287.2358 | 443479 | Abietane diterpenoids | Terpenoids |
| 25. | (2E)-3-[4-(sulfooxy)phenyl]prop-2-enoic acid | 20.79 | 289.0024 | 289.0012 | -4.01429 | [M+FA-H]- | 109.0278 | 6070438 | Cinnamic acids and derivatives | Shikimates and Phenylpropanoids |
| 26. | Allamandin | 18.47 | 289.0712 | 289.071 | -0.73632 | [M-H2O-H]- | 57.033, 109.0278, 203.0706 | 5281540 | Halogenated hydrocarbons | Fatty acids |
| 27. | Methyl 3-(2,3-dihydroxy-3-methylbutyl)-4-hydroxybenzoate | 25.96 | 293.0786 | 293.0776 | -3.34951 | [M+K]+ | 62.9824, 191.0448, 293.1715 | 11184361 | Shikimic acids and derivatives | Shikimates and Phenylpropanoids |
| 28. | Embelin | 28.17 | 293.1758 | 293.1785 | 9.095873 | [M-H]- | 96.9583, 293.1784 | 3218 | Benzoquinones | Polyketides |
| 29. | Limazepine C | 23.13 | 295.1053 | 295.1041 | -4.10152 | [M+Na]+ | 62.9823, 123.0416, 228.0149, 295.1039 | 42639506 | Benzodiazepine alkaloids | Alkaloids |
| 30. | Saussureolide | 23.53 | 297.1344 | 297.1336 | -2.56591 | [M-H]- | 71.0122,163.0597 | 1.01E+08 | Guaiane sesquiterpenoids | Terpenoids |
| 31. | Isotachioside | 17.03 | 301.0929 | 301.0926 | -0.96824 | [M-H]- | 123.0071 | 15098566 | NA | Shikimates and Phenylpropanoids |
| 32. | Dehydroabietic acid | 30.86 | 301.2162 | 301.2154 | -2.67626 | [M+H]+ | 71.0133, 177.0906, 301.2153 | 94391 | Abietane diterpenoids | Terpenoids |
| 33. | Sphinganine | 26.67 | 302.3054 | 302.3046 | -2.49909 | [M+H]+ | 88.076, 302.3045 | 91486 | Sphingoid bases | Fatty acids |
| 34. | Norlinoleic acid | 27.92 | 311.2228 | 311.2218 | -3.15271 | [M+FA-H]- | 74.023, 174.9547, 311.168 | 13932174 | Halogenated hydrocarbons | Fatty acids |
| 35. | Epicatechin | 19.49 | 313.0683 | 273.075 | -3.693 | [M+H-H2O]+ | 123.044, 273.1219 | 72276 | Flavan-3-ols | Shikimates and Phenylpropanoids |
| 36. | Xanthosine | 19.36 | 321.0243 | 321.0271 | 8.752858 | [M+K-2H]- | 67.3226, 174.9544, 275.0213 | 64959 | Purine nucleos(t)ides | Carbohydrates |
| 37. | 3,4-dihydroxy-5-(3,4,5-trihydroxybenzoyloxy)benzoic acid | 16.9 | 323.0398 | 323.0416 | 5.702425 | [M+H]+ | 174.9684 | 341 | Depsides | Polyketides |
| 38. | 8-Hydroxycannabinol | 30.99 | 325.1809 | 325.1834 | 7.630552 | [M-H]- | 88.0386,183.0108, 325.1837 | 44241652 | Cannabinoids | Polyketides |
| 39. | Leonuriside A | 17.12 | 331.1035 | 331.1031 | -1.07583 | [M-H]- | 67.3221,153.0178 | 14237626 | NA | NA |
| 40. | Zinolol | 11.92 | 332.134 | 332.1332 | -2.38648 | [M+H]+ | 108.0808, 152.0703, 314.1224 | 16216016 | NA | NA |
| 41. | Lipoxin B4 | 29.88 | 333.2066 | 333.2062 | -1.1528 | [M-H2O-H]- | 59.9837, 289.2169, 333.2065 | 5280915 | Halogenated hydrocarbons | Fatty acids |
| 42. | Atalantoflavone | 27.7 | 337.107 | 337.1037 | -9.93634 | [M+H]+ |  | 14162621 | Flavones | Shikimates and Phenylpropanoids |
| 43. | Melibiose | 5.22 | 341.1089 | 341.1078 | -3.32888 | [M-H]- | 59.0123, 89.0227, 179.0548 | 440658 | Disaccharides | Carbohydrates |
| 44. | Aknadicine | 17.88 | 346.1649 | 346.1634 | -4.32982 | [M+H]+ | 93.0369, 202.1069, 346.1634 | 442156 | Hasubanan alkaloids | Alkaloids |
| 45. | Scopolin | 4.69 | 353.0878 | 353.0886 | 2.248308 | [M-H]- | 71.0122, 115.0192, 191.0356, 263.0569, 353.0888 | 439514 | Simple coumarins | Shikimates and Phenylpropanoids |
| 46. | Blechnic acid | 20.43 | 357.0616 | 357.0581 | -9.77814 | [M-H]- | 109.0279, 203.0703 | 10316325 | Neolignans | Shikimates and Phenylpropanoids |
| 47. | Sucrose | 5.18 | 360.15 | 360.149 | -2.87244 | [M+NH4]+ | 85.0288, 145.0494 | 5988 | Disaccharides | Carbohydrates |
| 48. | Gibboside | 20.12 | 363.165 | 363.1615 | -9.52295 | [M+H]+ | Gibboside | 181392 | Iridoids monoterpenoids | Terpenoids |
| 49. | Isohumulone A | 29.24 | 363.2166 | 363.2132 | -9.36094 | [M+H]+ | 56.9654, 234.1221, 363.2132 | 93090 | Polyprenylated cyclic polyketides (Hop meroterpenoids) | Polyketides |
| 50. | Cellobiose | 5.18 | 365.1054 | 365.1043 | -3.09365 | [M+Na]+ | 203.0522, 365.1044 | 439178 | Disaccharides | Carbohydrates |
| 51. | Harpagide | 30.78 | 365.1442 | 365.144 | -0.61061 | [M+H]+ |  | 93045 | Iridoids monoterpenoids | Terpenoids |
| 52. | Blumeaene K | 22.38 | 365.1964 | 365.196 | -1.13188 | [M-H2O-H]- | 83.0485, 175.0388, 225.1279, 255.175, 303.1959 | 52936863 | Cadinane sesquiterpenoids | Terpenoids |
| 53. | 4-O-feruloyl-D-quinic acid | 4.69 | 367.1035 | 367.1041 | 1.753702 | [M-H]- | 71.0122, 115.019, 187.0407, 277.0729, 367.1039 | 10177048 | Cinnamic acids and derivatives | Shikimates and Phenylpropanoids |
| 54. | prostaglandin F2alpha dimethylamine | 30.86 | 368.3159 | 368.3148 | -3.04133 | [M+H]+ | 55.0549, 104.0707 | 5283101 | Halogenated hydrocarbons | Fatty acids |
| 55. | Patuletin | 6.83 | 369.0018 | 369.0049 | 8.337791 | [M+K-2H]- | 114.9499, 251.0142, 369.0035 | 5281678 | Flavonols | Shikimates and Phenylpropanoids |
| 56. | 1-O-p-Coumaroyl-beta-D-glucose | 20.87 | 371.0984 | 371.0976 | -2.07096 | [M+FA-H]- | 121.0277, 249.0611 | 14158116 | Cinnamic acids and derivatives | Shikimates and Phenylpropanoids |
| 57. | Arctigenin | 24.42 | 371.15 | 371.149 | -2.72795 | [M-H]- | 149.0593,191.0701 | 64981 | Dibenzylbutyrolactone lignans | Shikimates and Phenylpropanoids |
| 58. | Inophynone | 20.92 | 377.1747 | 377.177 | 6.004538 | [M+H]+ | 331.172, 377.177 | 5318443 | Pyranocoumarins | Shikimates and Phenylpropanoids |
| 59. | Betulalbuside A | 22.12 | 377.1817 | 377.1806 | -2.92758 | [M+FA-H]- | 67.3227, 146.9595, 174.9545, 208.0661 | 10382095 | Halogenated hydrocarbons | Fatty acids |
| 60. | Secoxyloganin | 21.77 | 385.1135 | 385.1133 | -0.44553 | [M-H2O-H]- | 67.3232, 112.9837, 251.5848 | 162868 | Secoiridoid monoterpenoids | Terpenoids |
| 61. | 21-Acetyloxy-17-hydroxypregna-1,4-diene-3,20-dione | 30.14 | 385.202 | 385.1986 | -8.95128 | [M-H]- | 69.0329, 273.2218 | 227110 | Pregnane steroids | Terpenoids |
| 62. | Geniposide | 16.67 | 387.1297 | 387.1293 | -0.95823 | [M-H]- | 67.3226, 225.0758 | 107848 | Iridoids monoterpenoids | Terpenoids |
| 63. | 9,13-Dihydroxy-4-megastigmen-3-one 9-glucoside | 21.32 | 387.2024 | 387.2017 | -1.91623 | [M-H]- | 71.0122, 152.0826, 225.1482, 387.1998 | 72791584 | Apocarotenoids(Îµ-) | Terpenoids |
| 64. | Kopetdaghin B | 30.18 | 387.253 | 387.2494 | -9.25896 | [M+H]+ | 166.0861, 387.2495 | 1.02E+08 | Halogenated hydrocarbons | Fatty acids |
| 65. | Monotropein | 18.91 | 389.1089 | 389.1081 | -2.14724 | [M-H]- | 59.0122, 97.0884, 138.0304, 174.9549 | 73466 | Iridoids monoterpenoids | Terpenoids |
| 66. | Secologanic acid | 21.45 | 397.1105 | 375.1253 | -8.34753 | [M+H]+ | 199.0937, 241.1041, 375.125 | 71607801 | Secoiridoid monoterpenoids | Terpenoids |
| 67. | evolvoid A | 22.74 | 397.1499 | 397.1495 | -0.89913 | [M-H2O-H]- | 71.0122, 132.02, 175.0387, 397.1496 | 16723783 | Cinnamic acids and derivatives | Shikimates and Phenylpropanoids |
| 68. | Picraquassioside A | 16.45 | 399.1286 | 399.1254 | -7.94954 | [M+H]+ | 67.3169, 161.0294, 399.1251 | 85502992 | NA | Shikimates and Phenylpropanoids |
| 69. | 2'-Methoxy-3-(2,4-dihydroxyphenyl)-1,2-propanediol 4'-glucoside | 16.67 | 405.1402 | 405.1396 | -1.56326 | [M+FA-H]- | 71.0122,177.0181, 346.1909 | 85301070 | NA | NA |
| 70. | Ginkgolide A | 20.25 | 407.1348 | 407.1339 | -2.10333 | [M-H]- | 69.033, 179.0335 | 9909368 | Prezizaane sesquiterpenoids | Terpenoids |
| 71. | Furcatin | 23.09 | 409.1499 | 409.1494 | -1.11717 | [M-H2O-H]- | 59.0123, 145.0279, 187.0388, 221.102, 342.0866, 409.1488 | 442789 | Cinnamic acids and derivatives | Shikimates and Phenylpropanoids |
| 72. | 8,9-Dihydro-5-hydroxy-8-(1-hydroxy-1-methylethyl)-6-(2-methyl-1-oxopropyl)-4-phenyl-2H-furo[2,3-h]-1-benzopyran-2-one | 26.67 | 409.1646 | 409.1611 | -8.4672 | [M+H]+ | 289.1039, 409.161 | 44257538 | Furocoumarins | Shikimates and Phenylpropanoids |
| 73. | trans-Ferulic acid | 18.87 | 411.105 | 411.0884 | -4.04656 | [2M+Na]+ | 140.068 | 445858 | Cinnamic acids and derivatives | Shikimates and Phenylpropanoids |
| 74. | Pteroside D | 21.27 | 411.2013 | 411.1975 | -9.348 | [M+H]+ | 411.1976 | 169738 | Illudalane sesquiterpenoids | Terpenoids |
| 75. | Isoreserpiline | 28.73 | 413.2071 | 413.2105 | 8.23316 | [M+H]+ | 105.0336, 163.0751, 413.2104 | 161345 | Corynanthe type | Alkaloids |
| 76. | 8-Hydroxyisocapnolactone-2',3'-diol | 20.79 | 421.114 | 421.1132 | -1.94387 | [M+FA-H]- | 71.0122, 93.0329, 113.0228,137.0228, 181.0494, 281.0662 | 12097137 | Simple coumarins | Shikimates and Phenylpropanoids |
| 77. | Glycyrrhizol A | 21.27 | 421.2009 | 421.2035 | 6.054141 | [M+H]+ | 421.203 | 11546269 | Coumestan | Shikimates and Phenylpropanoids |
| 78. | Hypericophenonoside | 23.05 | 425.1078 | 425.1069 | -2.20521 | [M+H]+ | 177.0543 | 1.02E+08 | Acyl phloroglucinols | Polyketides |
| 79. | Tortuoside | 22.38 | 425.1453 | 425.1446 | -1.69594 | [M-H]- | 161.0229, 425.1448 | 5491576 | Simple coumarins | Shikimates and Phenylpropanoids |
| 80. | 9-Hydroxy-4-methoxypsoralen 9-glucoside | 21.68 | 439.0882 | 439.0846 | -8.19373 | [M+FA-H]- | 121.0277, 174.9551, 249.0608 | 14034001 | Furocoumarins | Shikimates and Phenylpropanoids |
| 81. | sanguinolignan C | 16.4 | 441.1191 | 441.1163 | -6.36173 | [M-H]- | 89.0226, 341.1082 | 50908725 | Dibenzylbutyrolactone lignans | Shikimates and Phenylpropanoids |
| 82. | Lusitanicoside | 22.21 | 441.1766 | 441.1756 | -2.31461 | [M-H]- | 59.0123, 330.1313, 397.1833 | 442799 | Cinnamic acids and derivatives | Shikimates and Phenylpropanoids |
| 83. | Neoglabrescin B | 21.14 | 443.1923 | 443.1911 | -2.63748 | [M+FA-H]- | 71.0122, 443.1917 | 10992995 | Rhamnofolane diterpenoids | Terpenoids |
| 84. | 3-Methoxytyrosine | 28.9 | 445.1581 | 445.1731 | 3.361996 | [2M+Na]+ | 56.9654, 147.0326 | 1670 | Phenylethylamines | Alkaloids |
| 85. | Barlerin | 17.3 | 447.1508 | 447.1503 | -1.11891 | [M-H]- | 67.3226, 195.0648, 285.0973 | 162823 | Iridoids monoterpenoids | Terpenoids |
| 86. | Clusin | 30.14 | 447.1661 | 447.1689 | 6.364168 | [M+FA-H]- | 79.9556, 118.9644, 174.9547, 241.9738, 355.1589, 401.1638 | 44575537 | Arylnaphthalene and aryltetralin lignans | Shikimates and Phenylpropanoids |
| 87. | 6-Deoxodolichosterone | 30.86 | 449.3625 | 449.3589 | -8.09148 | [M+H]+ | 57.0705, 337.2336, 449.3587 | 13870426 | Cholestane steroids | Terpenoids |
| 88. | 8-C-p-Hydroxybenzylkaempferol | 18.47 | 451.1035 | 451.1023 | -2.55784 | [M+Hac-H]- | 57.0331, 109.0277, 203.0702, 245.081, 289.0711 | 44259046 | Flavonols | Shikimates and Phenylpropanoids |
| 89. | Garcimangosone D | 19.09 | 451.1246 | 451.1235 | -2.40113 | [M+Hac-H]- | 88.0385 | 11003703 | Acyl phloroglucinols | Polyketides |
| 90. | Myxopyroside | 16.09 | 451.1446 | 451.1413 | -7.35214 | [M+H]+ | 203.0521, 289.0886, 451.141 | 45360282 | Iridoids monoterpenoids | Terpenoids |
| 91. | Deoxynivalenol 3-glucoside | 20.7 | 457.1715 | 457.171 | -1.17192 | [M-H]- | 59.0123, 126.9996, 187.1114 | 71312510 | Trichothecane sesquiterpenoids | Terpenoids |
| 92. | Linalool 3,7-oxide beta-primeveroside | 21.85 | 463.2185 | 463.2176 | -1.91262 | [M-H]- | 71.0122,161.0439, 331.1755, 463.2186 | 1.32E+08 | Acyclic monoterpenoids | Terpenoids |
| 93. | Dehydrovomifoliol | 21.1 | 467.2404 | 467.2455 | 1.090046 | [2M+Na]+ | 122.0811 | 688492 | Apocarotenoids(Îµ-) | Terpenoids |
| 94. | Plumieride | 22.29 | 469.1352 | 469.1342 | -2.02556 | [M-H]- | 59.0123, 161.023, 425.1445 | 72319 | Iridoids monoterpenoids | Terpenoids |
| 95. | Reticulataxanthin | 30.09 | 473.3414 | 473.3436 | 4.633776 | [M+H]+ |  | 1.32E+08 | Halogenated hydrocarbons | Fatty acids |
| 96. | Kanokoside A | 19.9 | 475.1821 | 475.1807 | -2.94719 | [M-H]- | 71.0122, 89.0227, 149.0441, 180.0781, 211.0966 | 10457683 | Iridoids monoterpenoids | Terpenoids |
| 97. | 3'-O-Methyl-(-)-epicatechin 7-O-glucuronide | 21.41 | 479.1195 | 479.1183 | -2.50501 | [M-H]- | 71.0122, 122.0355, 311.0759 | 1.01E+08 | Flavan-3-ols | Shikimates and Phenylpropanoids |
| 98. | (all-E)-6'-Apo-y-caroten-6'-al | 29.37 | 487.3218 | 487.3208 | -1.98423 | [M+FA-H]- | 163.1113, 311.201, 487.3208 | 20055192 | Halogenated hydrocarbons | Fatty acids |
| 99. | Prespicatin | 21.54 | 497.1572 | 497.162 | 9.602777 | [M+K]+ | 393.1144, 497.1615 | 1.01E+08 | Halogenated hydrocarbons | Fatty acids |
| 100. | Steganacin | 4.95 | 501.1402 | 501.1451 | 9.711174 | [M+FA-H]- | 71.0122, 113.0224, 173.044, 441.1233 | 443021 | Dibenzocyclooctadienes lignans | Shikimates and Phenylpropanoids |
| 101. | 2,3-dihydro-3beta-methoxy withaferin A | 21.54 | 503.3003 | 503.305 | 9.27941 | [M+H]+ | 89.06 | 10767792 | Ergostane steroids | Terpenoids |
| 102. | Deacetylnomilinic acid | 26.67 | 508.2541 | 508.2531 | -1.97866 | [M+NH4]+ | 122.0812, 337.1588 | 13857951 | Limonoids | Terpenoids |
| 103. | Allanxanthone C | 23.09 | 509.2181 | 509.2226 | 8.857227 | [M+FA-H]- | 67.3232, 331.3314, 463.2152 | 1.36E+08 | Plant xanthones | Shikimates and Phenylpropanoids |
| 104. | Icariside II | 19.36 | 513.1766 | 513.1813 | 9.117426 | [M-H]- | 59.0123, 103.0383,341.1075 | 5488822 | Flavonols | Shikimates and Phenylpropanoids |
| 105. | Rubraflavone B | 23.58 | 513.2038 | 513.2065 | 5.295791 | [M+K]+ | 57.023, 513.206 | 44257580 | Flavones | Shikimates and Phenylpropanoids |
| 106. | Cofaryloside | 21.77 | 513.2705 | 513.2695 | -1.99024 | [M-H]- | 71.0122, 513.2701 | 1.02E+08 | Norkaurane diterpenoids | Terpenoids |
| 107. | Americanin B | 19.49 | 515.1313 | 515.1359 | 9.026166 | [M+Na]+ | 203.0522, 353.0833, 515.136 | 73352644 | Flavonolignans | Shikimates and Phenylpropanoids |
| 108. | Asystasioside A | 18.83 | 521.1876 | 521.1862 | -2.64732 | [M-H]- | 71.0123, 161.0441, 343.1387 | 1.02E+08 | Iridoids monoterpenoids | Terpenoids |
| 109. | Itoside G | 6.24 | 527.1548 | 527.1572 | 4.576189 | [M+H]+ | 203.052, 365.1044, 527.1569 | 25022462 | Simple phenolic acids | Shikimates and Phenylpropanoids |
| 110. | Pharboside E | 20.96 | 529.2654 | 529.2644 | -1.95771 | [M-H]- | 71.0122, 101.0226, 161.0439, 205.0342 | 45271592 | Norkaurane diterpenoids | Terpenoids |
| 111. | Nomilinic acid | 20.92 | 533.2381 | 533.2338 | -8.10771 | [M+H]+ | 551.3541 | 1.25E+08 | Limonoids | Terpenoids |
| 112. | Glabcensin A | 26.8 | 533.2392 | 533.2379 | -2.47806 | [M-H]- | 59.0123, 174.9548, 223.1127 | 10768649 | Kaurane and Phyllocladane diterpenoids | Terpenoids |
| 113. | Melissoidesin D | 22.38 | 541.2654 | 541.265 | -0.80155 | [M+FA-H]- | 59.0124 | 1.02E+08 | Kaurane and Phyllocladane diterpenoids | Terpenoids |
| 114. | Zeatin-9-glucoside-O-glucoside | 17.75 | 542.2104 | 542.2087 | -3.10007 | [M-H]- | 71.0122, 146.0805, 218.1021, 341.1089 | 1.73E+08 | Halogenated hydrocarbons | Fatty acids |
| 115. | Sibiricose A6 | 23.13 | 549.1814 | 549.1846 | 5.833573 | [M+H]+ | 56.9654, 146.9963, 389.1471, 503.1787 | 6326021 | NA | Shikimates and Phenylpropanoids |
| 116. | Viscumneoside VI | 23.09 | 551.1406 | 551.1372 | -6.21849 | [M+FA-H]- | 59.0123, 215.0313, 289.078 | 3082946 | Flavanones | Shikimates and Phenylpropanoids |
| 117. | Laccijalaric ester II | 30.35 | 551.3578 | 551.3541 | -6.79151 | [M+H]+ | 551.3541 | 1.02E+08 | Colensane and Clerodane diterpenoids | Terpenoids |
| 118. | 1-[[(4R,5S)-2-[(2R)-1-hydroxypropan-2-yl]-4-methyl-8-(4-methylpent-1-ynyl)-1,1-dioxo-4,5-dihydro-3H-6,1$l^{6},2-benzoxathiazocin-5-yl]methyl]-3-(4-methoxyphenyl)-1-methylurea | 29.71 | 556.2487 | 556.246 | -4.82006 | [M-H]- | 88.0387 | 54618931 | NA | Alkaloids |
| 119. | Basilol | 30.09 | 561.3938 | 561.3958 | 3.498186 | [M+H]+ | 393.2086, 561.3958 | 16655699 | Oleanane triterpenoids | Terpenoids |
| 120. | Epifisetinidol-(4beta->8)-catechin | 20.03 | 563.1548 | 563.1533 | -2.64162 | [M+H]+ | 127.0389, 287.0542 | 14332863 | Proanthocyanins | Shikimates and Phenylpropanoids |
| 121. | Croceaine A | 5.22 | 565.1594 | 565.1608 | 2.500887 | [M+K-2H]- | 61.9867, 223.0449, 258.1066 | 11329987 | Carboline alkaloids | Alkaloids |
| 122. | Proanthocyanidin A2 | 22.29 | 575.1195 | 575.1194 | -0.17422 | [M-H]- | 57.0331, 125.0224, 161.0226, 271.0241 | 124025 | Proanthocyanins | Shikimates and Phenylpropanoids |
| 123. | Genistein 7-O-(2-p-coumaroylglucoside) | 18.47 | 577.1352 | 577.1342 | -1.64651 | [M-H]- | 57.0331, 125.0227, 161.0228, 245.0211, 289.0711, 407.0762 | 1.32E+08 | Isoflavones | Shikimates and Phenylpropanoids |
| 124. | Terniflorin | 19.41 | 579.1497 | 579.1486 | -1.90324 | [M+H]+ | 68.9977, 127.0389, 287.0542, 409.0906 | 1.32E+08 | Flavones | Shikimates and Phenylpropanoids |
| 125. | Hydrangenoside E | 21.85 | 579.2447 | 579.2407 | -6.90677 | [M-H]- | 71.0122, 112.9839, 195.0651, 287.2009, 371.1825 | 21589718 | Secoiridoid monoterpenoids | Terpenoids |
| 126. | Cimilactone A | 22.07 | 591.3528 | 591.3574 | 7.84782 | [M+H]+ | 89.06 | 10908062 | Cycloartane triterpenoids | Terpenoids |
| 127. | Ourateaproanthocyanidin A | 19.67 | 593.1654 | 593.1644 | -1.60549 | [M+H]+ | 68.9977, 127.0389, 287.0545, 412.1446 | 9808627 | Flavan-3-ols | Shikimates and Phenylpropanoids |
| 128. | Tinyatoxin | 21.68 | 597.2494 | 597.2542 | 8.049954 | [M-H]- | 59.0123, 101.0228, 413.2696 | 76972186 | Daphnane diterpenoids | Terpenoids |
| 129. | Thermorubin A | 18.51 | 601.1341 | 601.1295 | -7.57269 | [M+H]+ | 102.0915, 449.083, 601.1298 | 5458199 | Halogenated hydrocarbons | Fatty acids |
| 130. | Viteoside A | 22.21 | 601.2866 | 601.2855 | -1.76824 | [M+FA-H]- | 59.0123, 161.0441 | 10769138 | Labdane diterpenoids | Terpenoids |
| 131. | Diosmin | 6.74 | 607.1668 | 607.1715 | 7.667892 | [M-H]- | 59.0123, 103.0019 | 5281613 | Flavones | Shikimates and Phenylpropanoids |
| 132. | Isovitexin 2''-O-arabinoside | 22.91 | 609.1461 | 609.1458 | -0.50326 | [M+FA-H]- | 67.3226, 300.0269 | 44468060 | Flavones | Shikimates and Phenylpropanoids |
| 133. | Vitexin 2''-O-rhamnoside | 23.62 | 623.1618 | 623.1611 | -1.05371 | [M+FA-H]- | 65.0017, 243.0287, 315.0504 | 5282151 | Flavones | Shikimates and Phenylpropanoids |
| 134. | O2-natafuranamine | 22.07 | 631.3144 | 631.3104 | -6.32848 | [M+K]+ | 56.9655, 277.0953, 585.278 | 53319040 | Steroidal alkaloids | Alkaloids |
| 135. | Tricetin 7,3'-diglucuronide | 19.27 | 635.0884 | 635.0915 | 4.820431 | [M-H2O-H]- | 57.033, 125.0227, 311.054, 451.0098 | 44258249 | Flavones | Shikimates and Phenylpropanoids |
| 136. | 1,2,6-Trigalloyl-beta-D-glucopyranose | 18.47 | 635.089 | 635.0914 | 3.798035 | [M-H]- | 125.0228, 295.0216 | 3357644 | Gallotannins | Shikimates and Phenylpropanoids |
| 137. | Hellicoside | 6.29 | 637.1769 | 637.1818 | 7.753452 | [M-H2O-H]- | 71.0122, 133.0126, 321.4103 | 5281778 | Cinnamic acids and derivatives | Shikimates and Phenylpropanoids |
| 138. | 6''-O-p-Coumaroyltrifolin | 31.16 | 639.1355 | 639.1324 | -4.91583 | [M+FA-H]- | 76.9684 | 12775850 | Flavonols | Shikimates and Phenylpropanoids |
| 139. | Alpindenoside A | 24.6 | 639.3022 | 639.3012 | -1.58858 | [M-H]- | 71.0123, 161.0439, 331.19 | 25158096 | Labdane diterpenoids | Terpenoids |
| 140. | Quercetin 3-(6''-p-hydroxybenzoylgalactoside) | 31.16 | 643.1305 | 643.1281 | -3.66402 | [M+Hac-H]- | 344.9954, 76.9683 | 91666354 | Flavonols | Shikimates and Phenylpropanoids |
| 141. | Taxuspine R | 22.47 | 653.2815 | 653.2784 | -4.71487 | [M-H]- | 59.0123 | 5321759 | Taxane diterpenoids | Terpenoids |
| 142. | Capsicum annuum Fluorescent chlorophyll catabolite | 22.74 | 665.2383 | 665.2426 | 6.455386 | [M+K-2H]- | 59.0123, 126.9995, 300.1834 | 15330925 | Halogenated hydrocarbons | Fatty acids |
| 143. | lumutinine B | 28.13 | 673.3748 | 673.3721 | -4.05722 | [M+H]+ | 69.0453, 177.088 | 56926390 | Carboline alkaloids | Alkaloids |
| 144. | Ajugaside A | 22.12 | 703.3183 | 703.3163 | -2.78289 | [M+FA-H]- | 71.022, 331.1914, | 10770608 | Totarane diterpenoids | Terpenoids |
| 145. | Thonningianin B | 18.02 | 721.141 | 721.1406 | -0.58724 | [M-H]- | 125.0226, 341.1259 | 10101397 | Chalcones | Shikimates and Phenylpropanoids |
| 146. | Pentandroside C | 28.9 | 743.4212 | 743.4167 | -6.09701 | [M+H]+ | 383.2028 | 1.01E+08 | Cholestane steroids | Terpenoids |
| 147. | Kaempferol 3-gentiobioside 7-rhamnoside | 21.68 | 755.204 | 755.201 | -3.99582 | [M-H]- | 300.0268 | 74428193 | Flavonols | Shikimates and Phenylpropanoids |
| 148. | Natsudaidain 3-(4-O-3-hydroxy-3-methylglutaroylglucoside) | 22.29 | 769.2197 | 769.2179 | -2.29508 | [M+FA-H]- | 65.0017, 314.0427 | 73092145 | Flavonols | Shikimates and Phenylpropanoids |
| 149. | Tenuifoliside C | 22.69 | 791.2369 | 791.2348 | -2.6505 | [M+Na]+ | 177.0544, 411.0887, 651.188, 790.4462 | 11968391 | NA | Shikimates and Phenylpropanoids |
| 150. | Hastatoside | 21.77 | 807.2564 | 807.2551 | -1.66881 | [2M-H]- | 71.0122, 193.0494 | 92043450 | Iridoids monoterpenoids | Terpenoids |
| 151. | Procyanidin C1 | 19.99 | 865.1985 | 865.1976 | -1.08456 | [M-H]- | 57.033, 125.0227, 289.0714, 407.0758 | 169853 | Proanthocyanins | Shikimates and Phenylpropanoids |
| 152. | 2''-O-p-Coumaroylvitexin | 18.47 | 1155.278 | 1155.273 | -3.9614 | [2M-H]- | 67.323, 125.0226, 161.0231, 289.0713, 407.0772 | 1.32E+08 | Flavones | Shikimates and Phenylpropanoids |

**Table S4** Summary of the 50 key differential metabolites, including fold change, FDR-adjusted p-values, VIP scores, and MS/MS match score

| **Name** | **PubChem ID** | **VIP score** | **Fold change** | **p-value** | **FDR adjusted p value** | **MS/MS score** |
| --- | --- | --- | --- | --- | --- | --- |
| Epicatechin | 72276 | 1.547386581 | 8.0041 | 0.0000672 | 0.0003433 | 7.8219 |
| Columbianetin | 92201 | 1.593510778 | 12.135 | 0.000059299 | 0.00021423 | 8.0599 |
| (S)-Pinocembrin | 68071 | 1.599533576 | 12.36 | 2.3236E-06 | 0.000012217 | 7.9778 |
| 4-ethenylphenyl cyclopenta-1,4-diene-1-carboxylate | 89042378 | 2.021926695 | 16.515 | 2.607E-10 | 1.6712E-08 | 7.2963 |
| Naringenin | 439246 | 1.600282277 | 12.369 | 3.5937E-06 | 0.000017915 | 8.4262 |
| Isotachioside | 15098566 | 1.041964816 | 5.2622 | 0.000030544 | 0.00011766 | 6.3466 |
| Scopolin | 439514 | 2.085775047 | -17.788 | 3.708E-10 | 2.0949E-08 | 7.538 |
| 21-Acetyloxy-17-hydroxypregna-1,4-diene-3,20-dione | 227110 | 1.056369932 | 5.4135 | 7.1234E-08 | 6.7012E-07 | 7.0219 |
| Tortuoside | 5491576 | 1.177612862 | 6.7019 | 0.000028546 | 0.00011069 | 8.0048 |
| Barlerin | 162823 | 1.009047425 | 4.9178 | 0.00010705 | 0.0003636 | 6.4758 |
| Garcimangosone D | 11003703 | 1.315881581 | 8.2008 | 0.00035714 | 0.0010952 | 7.0321 |
| Linalool 3,7-oxide beta-primeveroside | 131752012 | 1.359246873 | 8.9562 | 1.1058E-06 | 6.4105E-06 | 7.6709 |
| Allanxanthone C | 135513034 | 1.825751555 | 12.852 | 4.3036E-10 | 2.2532E-08 | 6.338 |
| Icariside II | 5488822 | 1.306251584 | 8.1407 | 0.00023003 | 0.00073312 | 6.5637 |
| Ajugaside A | 10770608 | 1.400001226 | 9.3379 | 0.00010175 | 0.00034786 | 6.0021 |
| Thonningianin B | 10101397 | 1.666104907 | 10.151 | 7.4532E-09 | 1.2986E-07 | 5.2417 |
| Hastatoside | 92043450 | 1.895929986 | 14.124 | 4.1029E-09 | 8.6659E-08 | 6.4931 |
| Blechnic acid | 10316325 | 1.866431107 | 13.492 | 0.0035956 | 0.0089755 | 6.9795 |
| evolvoid A | 16723783 | 1.110276811 | 5.8687 | 0.00083832 | 0.002387 | 7.5296 |
| Ginkgolide A | 9909368 | 1.854272869 | 13.373 | 1.9205E-08 | 2.527E-07 | 6.4954 |
| Clusin | 44575537 | 1.970546131 | 15.518 | 1.9258E-11 | 3.8617E-09 | 6.5486 |
| Steganacin | 443021 | 1.283030189 | 7.9434 | 0.000018758 | 0.0000765 | 6.8822 |
| Pharboside E | 45271592 | 1.129209074 | 6.1746 | 4.0292E-06 | 0.000019761 | 7.6945 |
| Glabcensin A | 10768649 | 1.875717535 | 13.761 | 1.851E-08 | 2.4614E-07 | 6.0031 |
| Zeatin-9-glucoside-O-glucoside | 172642872 | 1.044276374 | 5.2811 | 0.000013226 | 0.000055949 | 6.2906 |
| Viscumneoside VI | 3082946 | 1.160642311 | 6.5236 | 0.000017807 | 0.000072918 | 6.7226 |
| Epifisetinidol-(4beta->8)-catechin | 14332863 | 1.880144824 | 13.829 | 7.8616E-12 | 2.4028E-09 | 6.8599 |
| Proanthocyanidin A2 | 124025 | 1.164019749 | 6.5751 | 1.7223E-08 | 2.3497E-07 | 7.1619 |
| Genistein 7-O-(2-p-coumaroylglucoside) | 131752444 | 1.330754062 | 8.5921 | 1.2031E-11 | 3.0978E-09 | 7.3661 |
| Tinyatoxin | 76972186 | 1.266984754 | 7.7941 | 9.099E-08 | 8.1459E-07 | 5.9612 |
| Alpindenoside A | 25158096 | 1.137520248 | 6.2488 | 0.00008004 | 0.00028035 | 6.3514 |
| Taxuspine R | 5321759 | 1.044922757 | 5.2827 | 0.00003622 | 0.00013746 | 6.0699 |
| Procyanidin C1 | 169853 | 1.441061387 | 10.065 | 2.3402E-07 | 1.7742E-06 | 6.8005 |
| 2''-O-p-Coumaroylvitexin | 131752196 | 1.866449638 | 13.586 | 3.8297E-09 | 8.2359E-08 | 6.3172 |
| lumutinine B | 56926390 | 1.669686972 | -10.245 | 4.6737E-07 | 3.1085E-06 | 6.8736 |
| Plumieride | 72319 | 2.291936086 | 14.479 | 3.71E-09 | 0.000000105 | 7.254 |
| 3,4-dihydroxy-5-(3,4,5-trihydroxybenzoyloxy)benzoic acid | 341 | 1.046869543 | -3.7113 | 0.0000149 | 0.0000922 | 6.124 |
| Myxopyroside | 45360282 | 1.109552121 | 4.1396 | 0.00046612 | 0.0018979 | 5.5229 |
| Plumieride | 72319 | 2.291936086 | 14.479 | 3.71E-09 | 0.000000105 | 7.254 |
| Americanin B | 73352644 | 1.485090619 | 7.4667 | 0.00000176 | 0.0000145 | 7.0306 |
| Sibiricose A6 | 6326021 | 1.388776548 | 6.4235 | 0.00039592 | 0.0016401 | 6.9453 |
| Epifisetinidol-(4beta->8)-catechin | 14332863 | 2.193010348 | 12.97 | 5.4E-11 | 8.44E-09 | 7.4692 |
| Thermorubin A | 5458199 | 1.507731306 | 7.7005 | 0.000000014 | 0.000000268 | 5.9294 |
| Pentandroside C | 101361933 | 1.007128016 | 3.4324 | 0.0000396 | 0.0002163 | 7.2686 |
| Procyanidin C1 | 169853 | 1.712200448 | 9.7808 | 0.0000696 | 0.0003548 | 7.5783 |
| Abietinal | 443479 | 1.241802333 | 5.228 | 0.00000308 | 0.0000235 | 8.0716 |
| Dehydroabietic acid | 94391 | 1.225727958 | 5.0852 | 0.0000128 | 0.0000809 | 8.0774 |
| Nomilinic acid | 124708033 | 2.236742522 | 13.557 | 0.0035462 | 0.011169 | 5.8218 |
| Terniflorin | 131752172 | 1.722189455 | 10.01 | 0.00000289 | 0.0000222 | 8.2948 |
| Ourateaproanthocyanidin A | 9808627 | 1.172312423 | 4.5827 | 0.0018274 | 0.0062409 | 7.2469 |

**Table S5** Quality assessment of the subtracted genome using QUAST software

| **Attributes** | ***Alpinia galanga*** |
| --- | --- |
| Contigs selected | 3983 |
| Contigs ( >= 0 bp) | 20664 |
| Contigs ( >= 1000 bp) | 1413 |
| Contigs ( >= 5000 bp) | 141 |
| Contigs ( >= 10000 bp) | 92 |
| Contigs ( >= 25000 bp) | 32 |
| Contigs ( >= 50000 bp) | 8 |
| Largest contig | 88956 |
| Total length | 6784889 |
| Total length ( >= 0 bp) | 10231351 |
| Total length ( >= 1000 bp) | 4804394 |
| Total length ( >= 5000 bp) | 2739863 |
| Total length ( >= 10000 bp) | 2398728 |
| Total length ( >= 25000 bp) | 1440495 |
| Total length ( >= 50000 bp) | 559945 |
| N50 | 2089 |
| N75 | 918 |
| L50 | 357 |
| L75 | 1711 |
| GC (%) | 42.57 |

**Table S6** Details of selected species specific DNA markers

| **Plant Name** | **Primer** | **Sequence (5'-3')** | **Primer length (bp)** | **Amplicon Size (bp)** | **GenBank Accession number** |
| --- | --- | --- | --- | --- | --- |
| *A. galanga* | Forward | CCATTGCAAACCCTCAAACT | 20 | 612 | PP503004 |
|  | Reverse | ATTACTGCCGGATCTTGTGG |  |  |  |

**Table S7 Details of DNA barcoding of selected raw drug samples using universal DNA markers**

| **Sample** | **nBLAST output** | **Query coverage** | **Percent identitiy** | **Marker used** |
| --- | --- | --- | --- | --- |
| Rasna 1 | *Pluchea lanceolata* | 100 | 87.1 | *nrITS* |
| Rasna 2 | *Tribulus eichlerianus* | 100 | 99.8 | *nrITS* |
| Rasna 3 | *Bergenia ciliata* | 100 | 98.87 | *nrItS* |

**Supplementary Figures**

**Fig. S1** Herbarium voucher specimens authenticated by a certifies taxonomist of A) *Alpinia galanga*, B) *Alpinia calcarata*

**Fig. S2** Photographs of germplasms of selected plants preserved at our greenhouse: A) *Alpinia galanga,* B) *Alpinia calcarata* MSLS, MAHE, Manipal

**Fig. S3** A) Electrophoretic image of PCR with *nrITS* for fresh samples. Lane 1: 100 bp DNA ladder, 2: *A.calcarata,* 3: *A.galanga,* 4: NC-Negative control. B) and C) ClustalW alignment of sequences matched with NCBI database.

**Fig. S4.** Phylogenetic construction of Alpinia spp., based on *nrITS* sequences with RaxML v8.0 software. Bootstrap values (%) are shown at the corresponding nodes.

**Fig. S5.** Total ion chromatograms of *A. galanga* in triplicates under ESI positive and negative polarity switching modes

**Fig. S6.** Total ion chromatograms of *A. calcarata* in triplicates under ESI positive and negative polarity switching modes

**Fig. S7.** Multivariate statistical analysis plots for LC-MS data of *A. calcarata vs A. galanga*: (A) OPLS‒DA plot (B) OPLS‒DA model overview in positive ionisation mode (C) Cross-validation plots (D) OPLS‒DA plot (E) OPLS‒DA model overview (F) Cross-validation plots in negative ionisation mode**.**
