# Supplementary material for: A genomic approach for accurate identification and authentication of Alpinia galanga (L.) Willd. using SSH and NGS technologies
Source: Physiol Mol Biol Plants. 2026 Oct 12;32(11):2479–91. doi: 10.1007/s12298-026-01827-y (PMC13759926; doi:10.1007/s12298-026-01827-y)
Supplement: Supplementary file 2 [file 12298_2026_1827_MOESM3_ESM.docx]

**Uncropped electrophoretic gel images**

**Uncropped electrophoretic image of Figure 6.** Validation of the DNA marker (AG_SP1) of A. galanga. (A) Lanes 1–6: A. galanga samples, 7: 100 bp DNA ladder, 8–13: A. calcarata samples, 14: NC - negative control

**Uncropped electrophoretic image of Figure 7.** Validation of DNA marker (AG_SP1) of raw drug samples with AG_SP1, Lane 1: Rasna 1, 2: Rasna 2, 3:Rasna 3, 4: PC - positive control, 5: NC-Negative control.

**Uncropped electrophoretic image of Figure S5.** Electrophoretic image of PCR with *nrITS* for fresh samples. Lane 1: 100 bp DNA ladder, 2: *A.calcarata,* 3: *A.galanga,* 4:NC-Negative control.
